# Identification of Secreted Exoproteome Fingerprints of Highly-Virulent and Non-Virulent *Staphylococcus aureus* Strains

**DOI:** 10.3389/fcimb.2016.00051

**Published:** 2016-05-06

**Authors:** Emilia Bonar, Iwona Wojcik, Urszula Jankowska, Sylwia Kedracka-Krok, Michal Bukowski, Klaudia Polakowska, Marcin W. Lis, Maja Kosecka-Strojek, Artur J. Sabat, Grzegorz Dubin, Alexander W. Friedrich, Jacek Miedzobrodzki, Adam Dubin, Benedykt Wladyka

**Affiliations:** ^1^Department of Analytical Biochemistry, Faculty of Biochemistry, Biophysics and Biotechnology, Jagiellonian University Krakow, Poland; ^2^Malopolska Centre of Biotechnology, Jagiellonian University Krakow, Poland; ^3^Department of Physical Biochemistry, Faculty of Biochemistry, Biophysics and Biotechnology, Jagiellonian University Krakow, Poland; ^4^Department of Microbiology, Faculty of Biochemistry, Biophysics and Biotechnology, Jagiellonian University Krakow, Poland; ^5^Department of Veterinary and Animal Reproduction and Welfare, Faculty of Animal Welfare, University of Agriculture in Krakow Krakow, Poland; ^6^Department of Medical Microbiology, University of Groningen, University Medical Center Groningen Groningen, Netherlands

**Keywords:** chicken embryo model, pathogen, protein, proteomics, Staphylococcus, virulence

## Abstract

*Staphylococcus aureus* is a commensal inhabitant of skin and mucous membranes in nose vestibule but also an important opportunistic pathogen of humans and livestock. The extracellular proteome as a whole constitutes its major virulence determinant; however, the involvement of particular proteins is still relatively poorly understood. In this study, we compared the extracellular proteomes of poultry-derived *S. aureus* strains exhibiting a virulent (VIR) and non-virulent (NVIR) phenotype in a chicken embryo experimental infection model with the aim to identify proteomic signatures associated with the particular phenotypes. Despite significant heterogeneity within the analyzed proteomes, we identified alpha-haemolysin and bifunctional autolysin as indicators of virulence, whereas glutamylendopeptidase production was characteristic for non-virulent strains. Staphopain C (StpC) was identified in both the VIR and NVIR proteomes and the latter fact contradicted previous findings suggesting its involvement in virulence. By supplementing NVIR, StpC-negative strains with StpC, and comparing the virulence of parental and supplemented strains, we demonstrated that staphopain C alone does not affect staphylococcal virulence in a chicken embryo model.

## Introduction

Staphylococci constitute an important component of physiological biocenosis of skin and nose vestibule mucous membranes in man and anterior nares in animals (Devriese, 1990; Chen and Tsao, 2013). However, staphylococci are also dangerous opportunistic pathogens responsible for multiple infections (Bohach et al., 1990; Lowy, 1998). Among many species of staphylococci, *Staphylococcus aureus* receives major attention as an etiologic factor of human and livestock disease with rapidly increasing antibiotic resistance (Chambers and Deleo, 2009).

Recent data demonstrates that although *S. aureus* infects many different host species, particular strains are adapted to certain hosts only (McCarthy et al., 2014). Species-specific adaptation is associated with acquisition and/or loss of mobile genetic elements (MGEs) (Malachowa and DeLeo, 2010; Lindsay, 2014) but also with reorganization of the entire genomes (Sung et al., 2008; Smyth et al., 2009; Hata et al., 2010). In line with the above, our previous study demonstrated that clear differences in virulence between avian strains as evaluated in a chicken embryo model are not reflected in a nematode model where generally low virulence and only minor differences between the evaluated strains were observed. Moreover, the significant differences in virulence in the embryo model correlated with the strain genotype, which was not the case in the nematode model (Polakowska et al., 2012). This suggests species-specific adaptations in the repertoire of virulence determinants. Several studies provide examples of host specific adaptations, including the arginine catabolic mobile element (ACME), which enhances survival in the human host (Diep et al., 2006, 2008; Barbier et al., 2010) or additional ruminant and horse-specific alleles of the von Willebrand factor-binding protein (Viana et al., 2010) modulating virulence specifically in these species. It has also been shown that human-to-poultry host specificity jump was associated with pseudogenization of the *spa* gene and acquisition of avian specific MGEs, among others a 17-kb pAvX plasmid encoding a thiol protease StpC (Takeuchi et al., 1999, 2002; Lowder et al., 2009). However, since host adaptation is clearly reflected in the organization of the entire genomes (Sung et al., 2008; Smyth et al., 2009; Hata et al., 2010), many adaptive processes are yet to be discovered.

Most of the identified host specific adaptations and virulence determinants are associated with the plasticity of the extracellular proteome, the primary site of host-pathogen interaction. However, despite many years of research effort and a few successful examples, we still poorly understand the role of particular exoproteins. The first major reason is the high genetic variability among staphylococci (Moore and Lindsay, 2001), suggesting that in most cases certain combinations of multiple factors rather than the expression of a particular one are involved in virulence, while there were no tools to analyze such complex systems until relatively recently (Becker and Bubeck Wardenburg, 2015). The second key reason is that the research is mostly focused on the interactions with the human host, while perforce animal models are used to verify the role of particular factors resulting in contradictory observations (Melehani et al., 2015; Shukla et al., 2015; Spaan et al., 2015).

In this study, we therefore chose to overcome these major issues by applying a holistic proteomic approach to identify virulence signatures within extracellular proteomes of avian strains carefully characterized in terms of their pathogenic potential in a chicken embryo model.

## Materials and methods

### Bacterial strains and growth conditions

The exoproteome of poultry-derived *S. aureus* strains exhibiting either high (CH3, CH5, CH9, CH23, and PA2) or low (ph1, ch22, pa3, ch24, ph2) virulence in a chicken embryo experimental infection model was analyzed. The detailed genetic characteristics, including sequence types (ST), *agr* status, phylogenetic relationships, and virulence data concerning the strains used in this study were described previously (Polakowska et al., 2012). *S. aureus* strains RN4220 (Kreiswirth et al., 1983), Newman (Duthie and Lorenz, 1952), ph1, and ch24 carrying a pALCP2 control plasmid (Bukowski et al., 2013) or a pALCP2/stpC plasmid driving the expression of a cysteine protease, staphopain C (StpC), were obtained by electroporation. Characteristics of the strains used is shown in Table 1.

**Table 1:** **Genetic and phenotypic characteristics of *S. aureus* strains used in the study**.

**Strain**	**MLST type**	***spa* type**	***agr* group**	***agr* status^a^**	**Virulence in chicken embyo model^b^**	**Proteolysis**	**Haemolysis**
CH3	5	t002	II	Pos	yes	+	++
CH5	5	t002	II	Pos	yes	+	++
CH9	5	t002	II	Pos	yes	+++	−
ch22	5	t002	II	Neg	no	+++	+
CH23	5	t3478	II	Pos	yes	+++	++
ch24	1	t002	III	Pos	no	+	+
ch24/pALCP2	1	t002	III	ND	^c^	+	ND
ch24/pALCP2/stpC	1	t002	III	ND	^c^	+++	ND
Newman/pALCP2	2125	t008	I	ND	^c^	−	ND
Newman/pALCP2/stpC	2125	t008	I	ND	^c^	+++	ND
PA2	1346	t002	II	Pos	yes	+++	+
pa3	692	t8646	I	Pos	no	−	−
ph1	1347	t8646	I	Pos	no	−	−
ph1/pALCP2	1347	t8646	I	ND	^c^	−	ND
ph1/pALCP2/stpC	1347	t8646	I	ND	^c^	++	ND
ph2	692	t8646	I	Pos	no	+++	−
RN4220/pALCP2	8	t211	I	ND^d^	^c^	−	ND
RN4220/pALCP2/stpC	8	t211	I	ND^d^	^c^	+++	ND

a *Defined as an ability to produce δ-haemolysin assayed according to Traber et al. (2008); neg, negative; pos, positive; ND, not determined; see also S3 Figure;*

b *according to Polakowska et al. (2012);*

c *see Figure **2** in Results section;*

d *RN4220 displays agr negative phenotype according Traber and Novick (2006); −, lack, +, weak, ++, moderate, +++, strong activity*.

For exoproteome analysis, the bacteria were cultured in tryptic soy broth (TSB) for 16 h at 37°C with vigorous shaking. Supernatants were collected by centrifugation (30 min, 15,000 g, 4°C) and decanting, repeated twice to ensure disposal of bacterial cells. For assessment of virulence, the bacteria were prepared as described previously (Polakowska et al., 2012), save that the media were supplemented with chloramphenicol (10 μg/ml) to ensure pALCP2 plasmid maintenance.

The cultures were conducted in three biological replicates for each strain in the same experimental conditions.

### Proteomic analysis

**Figure 1:**
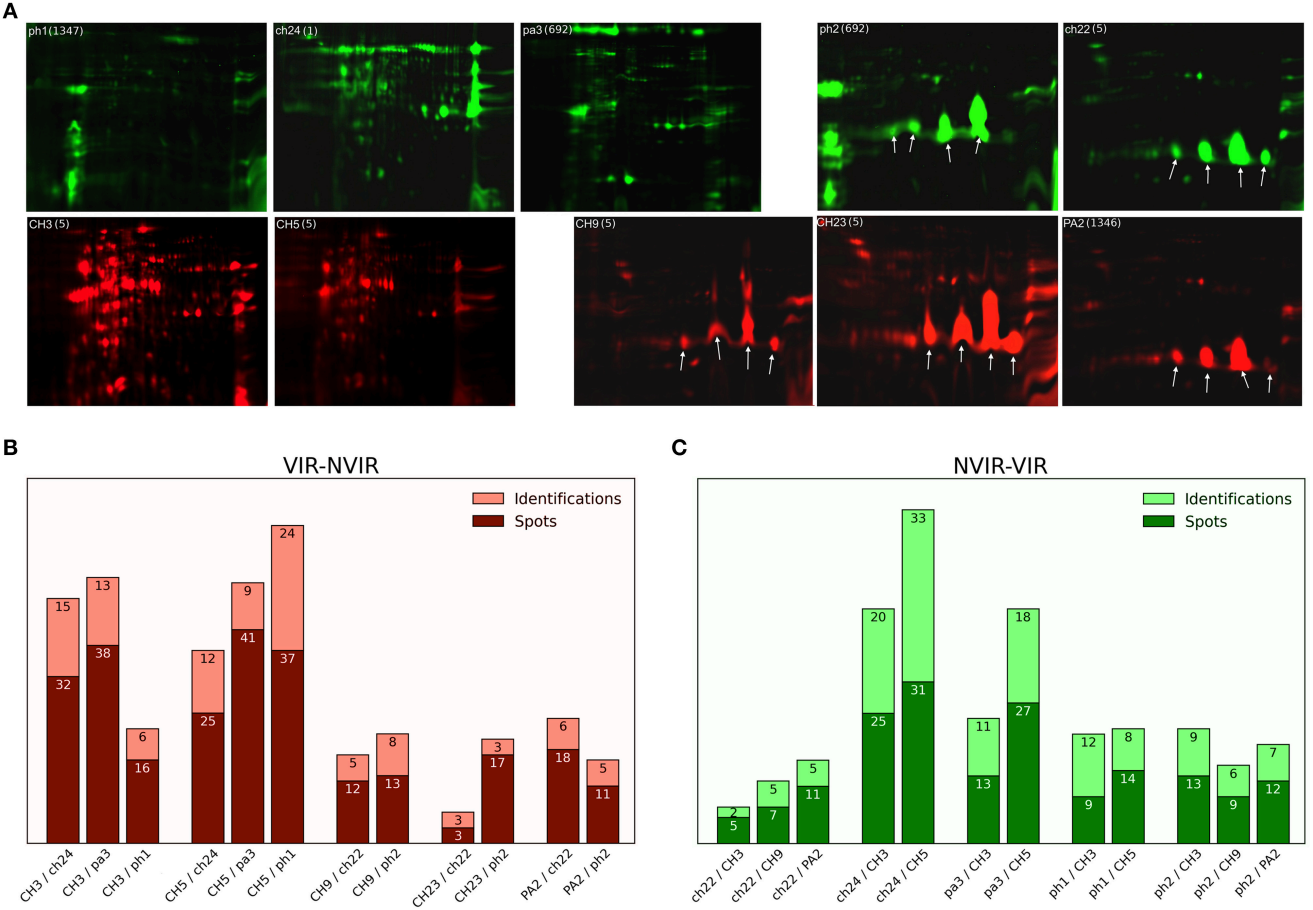
**Staphylococcal exoproteome profiles analyzed by 2D gel electrophoresis and mass spectrometry (MS). (A)** The exoproteomes of poultry-derived *S. aureus* strains exhibit two major types of profiles: (right) dominated by staphopain C (StpC; arrows) expression or (left) lacking the expression of staphopain C. The profile type does not correlate with virulence in the chicken embryo model—virulent strains CH3, CH5, CH9, CH23, PA2 (red); non-virulent strains ph1, ch22, pa3, ch24, ph2 (green). Molecular weight range is 6–116 kDa, pI range is 3–10 for gels without staphopain C (CH3, CH5, ph1, ch24, and pa3) and from 4 to 7 in case of gels with StpC (CH9, CH23, PA2, ph2, and ch22). Sequence type (ST) is provided next to the strain tag in a bracket. **(B,C)** respectively, summary of 2D DIGE and MS identification analyses of the staphylococcal exoproteomes of the virulent and non-virulent strains (dark color bar—number of differentiating spots cut out from the gel, light color bar—number of proteins identified by MS analysis; the differences in the above referenced numbers arise mainly from the fact that in many cases multiple spots originated from a single protein).

Cleared culture supernatants were incubated with an equal volume of 20% (w/v) trichloroacetic acid (TCA) in acetone at −20°C for 24 h. Precipitated proteins were recovered by centrifugation (25 min, 18,000 g, 4°C) and the resulting pellet was washed tree times with ice-cold acetone and air dried. The samples were dissolved in lysis buffer (30 mM Tris, 7 M urea, 2 M thiourea, 4% CHAPS) and the total protein concentration was quantified using Quick Start Bradford reagent (Biorad). The protein samples from the virulent and non-virulent strains as well as the internal standard, prepared by combining the protein samples from all tested replicates of virulent and non-virulent strains, were labeled with spectrally resolvable fluorescent dyes (Cy3, Cy5, and Cy2, respectively; GE Healthcare). The exoproteomes were compared within 12 pairs of virulent and non-virulent strains as summarized in Figure 1. The labeled samples of exo-proteins from the compared virulent strain, non-virulent strain, and the internal standard were combined and subjected to two-dimensional difference gel electrophoresis (2D DIGE) (Alban et al., 2003; Timms and Cramer, 2008; Minden et al., 2009). For each pair of the analyzed strains, in total 45 μg of fluorescently labeled proteins were loaded on 7 cm immobilized pH gradient strips (IPG) by in-gel rehydratation. Isoelectrofocusing (IEF) was carried out using Protean IEF Cell (Bio-Rad), while proteins were separated in the second dimension in 12% acrylamide gel according to the Laemmli method (Laemmli, 1970). The gels were scanned using Typhoon Trio + (GE) and gel images were analyzed with Image Quant v.7.0 and DeCyder 2D software v.7.2 (GE). Subsequently, the gels were silver stained (Shevchenko et al., 1996) and the differentiating spots were excised and identified using mass spectrometry.

### Mass spectrometry identification of proteins

The excised gel fragments were destained by several subsequent washes in 25 and 50% (v/v) acetonitrile (ACN) in 25 mM ammonium bicarbonate buffer (NH_4_HCO_3_), pH 8.0 at 37°C. Next, the gel fragments were dehydrated in ACN, dried using a vacuum concentrator, and rehydrated using 15 μL of trypsin (Biocentrum) solution (10 ng/μL in 25 mM NH_4_HCO_3_, pH 8.0) for 15 min. Additional 20 μL of 25 mM NH_4_HCO_3_ buffer were added and digestion was carried out overnight at 37°C. Peptides were extracted by sonication and dehydrated in ACN. The extracts were evaporated to dryness using a vacuum concentrator and suspended in 2% (v/v) ACN in water containing 0.05% (v/v) trifluoroacetic acid (TFA). The resulting peptides were separated on a 15 cm × 75 μm RP column (2 μm, Acclaim PepMap 75 μm 100 Å Nano Series TM Column) using a 2–40% gradient of ACN in 0.05% formic acid during 30 min on UltiMate 3000RS LCnanoSystem (Dionex). The system additionally contained a C_18_ precolumn (3 μm, 2 cm × 75 μm Acclaim PepMap Nano trap Column). The peptides were analyzed on a coupled MicrOTOF-QII mass spectrometer (Bruker) equipped with an Appollo Source ESI nano-sprayer with a low-flow nebulizer. The MS was operated in the standard data-dependent acquisition MS/MS mode with fragmentation of most intensive precursor ions.

### Analysis of mass spectrometry data

MS spectra were recalibrated using fragment ions of trypsin-derived peptides. Raw data was pre-processed with Data Analysis 4.0 software (Bruker, Germany) into the Mascot Generic format. The SwissProt_201202 non-redundant protein database taxonomically restricted to *Firmicutes* (gram-positive bacteria; 68 048 sequences) was queried with the obtained peak lists using an in-house Mascot server (v.2.3.0, Matrix Science, London, UK). The following search parameters were applied: permitted number of missed cleavages—one, fixed modification—carbamidomethylation (C), variable modification—oxidation, protein mass—unrestricted, peptide mass tolerance—±20 ppm, fragment mass tolerance—±0.05 Da. Only identifications with a score value over 100 were considered relevant for further analysis. If more than one protein was identified in one spot, only those with the scores of over 50% of the highest scoring protein were considered in further analysis.

### Virulence assessment in a chicken embryo model

The experiments were performed in compliance with the animal protection laws of Poland. Experiments using chicken embryos were terminated on development day 17 at the latest, 4 days prior to hatching. The virulence of each tested strain was assessed as described previously (Polakowska et al., 2012). In brief, a titrated suspension of bacteria of a tested strain was inoculated to embryos at the 10th day of their development and then the viability of embryos was monitored for the next 7 days by candling. All experiments were repeated in three independent runs. In each run, the effect of each strain was evaluated at two dilutions: 10^6^ and 10^4^ CFU/egg. Each run involved 20 eggs for each bacterial dilution and the control.

## Results

### The extracellular proteomes of virulent and non-virulent *S. aureus* strains are highly heterogeneous

We have recently demonstrated a clear correlation between the genotype and the virulence level as evaluated in the chicken embryo model within a collection of poultry-derived *S. aureus* strains (Polakowska et al., 2012). In this study, we intended to elucidate how the observed genetic differences determining the virulence level translated into differences within respective extracellular proteomes. To this end, of the strains characterized in our previous study, five virulent (VIR) ones were chosen (CH3, CH5, CH9, CH23, and PA2) and their extracellular proteomes were analyzed and compared to those of the five previously characterized non-virulent (NVIR) strains (ph1, ch22, pa3, ch24, and ph2). The extracellular proteomes of all tested strains were analyzed by two-dimensional polyacrylamide gel electrophoresis (2D-PAGE) demonstrating significant variability both between the VIR and NVIR groups and within each group (results not shown). To identify the components of the extracellular proteome possibly involved in virulence, we paired the VIR strains with those belonging to the NVIR group according to the overall similarities within the electrophoretic patterns and compared their secreted proteomes using difference gel electrophoresis (DIGE). Twelve pairs were analyzed in total allowing comparison of each virulent strain with either two or three non-virulent counterparts (Figure 1, S1, S2 Tables). Analysis of DIGE gel images confirmed high variation in the number and location of differentiating protein spots among the virulent strains (from 20 spots identified in CH23, paired with ch22 and ph2, up to 103 spots for CH5, paired with pa3, ph1, and ch24; Figure 1B). Comparable variation was observed for the non-virulent strains (from 23 spots in ph1 to 56 in ch24, both paired with CH3 and CH5; Figure 1C). As in case of the high variability in the electrophoretic patterns of the analyzed exoproteomes, the number of protein spots differentiating the VIR and NVIR exoproteomes in the analysis of particular pairs was also highly variable, ranging from eight spots differentiating the proteomes of CH23/ch22 up to 64 spots in the CH5/pa3 pair, even despite the fact that, prior to comparative analysis, the strains were paired based on the overall similarity of respective electrophoretic patterns. Interestingly, a majority of the identified differences in the exoproteomes of the VIR and NVIR strains were of a qualitative nature. Moreover, the total number of spots identified as differentiating in all the VIR strains (263) was substantially higher than of those in the NVIR strains (176).

### Staphopain C overexpression does not affect the virulence level

**Figure 2:**
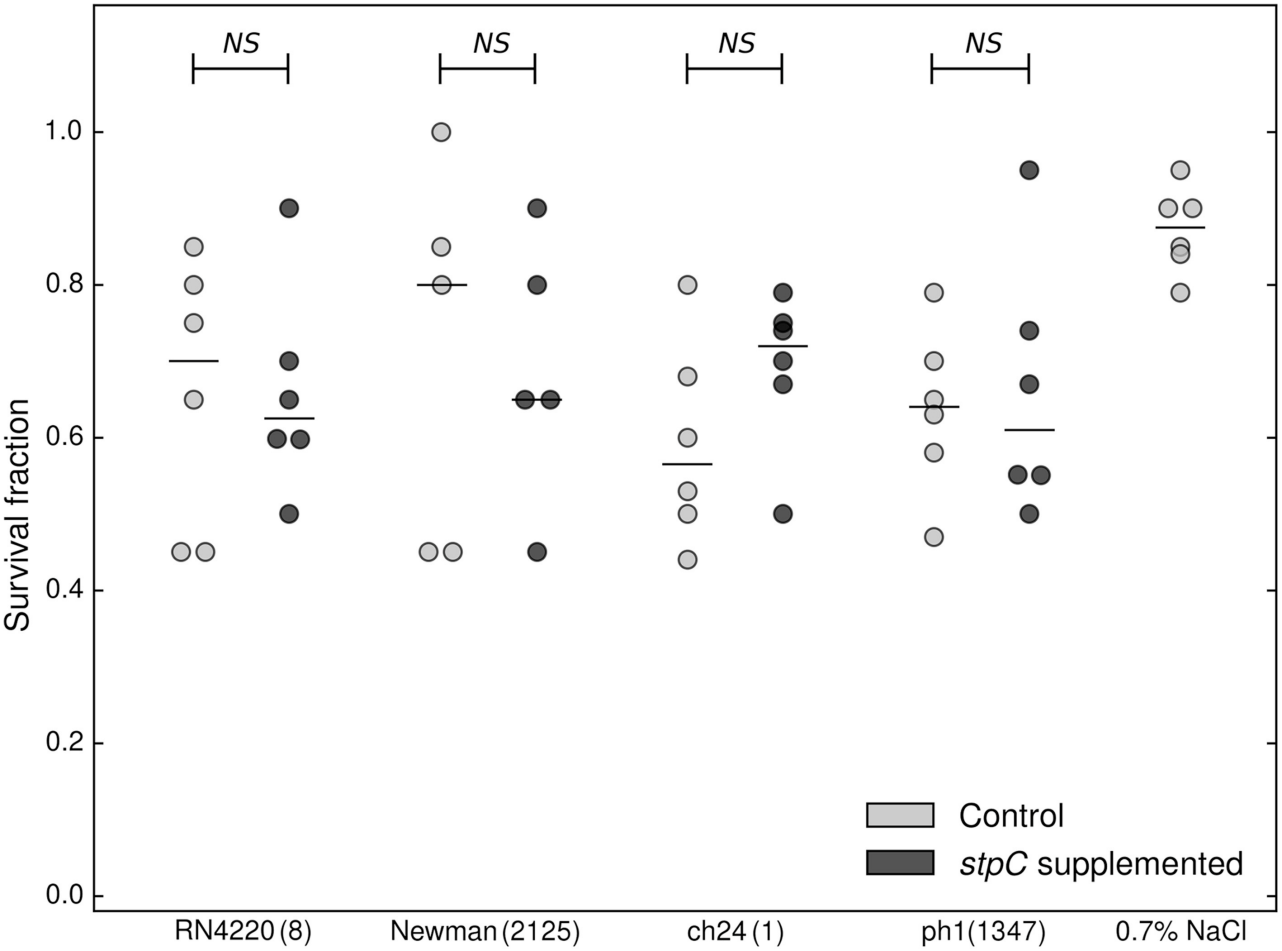
**Comparison of virulence of WT (stpC^−^, supplemented with a control plasmid) and staphopain C-supplemented staphylococcal strains in the chicken embryo model**. Cumulative survival of chicken embryos 7 days following inoculation with staphylococcal strains supplemented with staphopain C expressing plasmid (dark gray circles), and control plasmid (light gray circles). The median is marked with a horizontal line. Each circle corresponds to an independent data point. No statistically significant differences were recorded (Mann-Whitney test). ST is provided next to the strain tag in a bracket.

One striking finding of the electrophoretic analysis reported above was that the exoproteomes of certain strains are characterized by four major spots almost exclusively dominating the electrophoretic pattern, which indicates strong overexpression of those proteins. LC-MS/MS analysis demonstrated that all those spots originate from a single protein—a cysteine protease, staphopain C (StpC) (Figure 1A). The protease is encoded in the pAvX plasmid, which is a part of the poultry-specific pool of accessory genetic material, and has been suggested to play a role in poultry dermatitis (Kuramasu et al., 1967; Takeuchi et al., 2002; Lowder et al., 2009). Interestingly, however, we observed StpC overexpression in strains belonging to both VIR (CH9, CH23, and PA2) and NVIR (ph2 and ch22) groups. To analyze the discrepancy between the above data and our own observations, we verified the influence of StpC on the virulence of different strains in our chicken embryo model. To this end, the non-virulent strains, including RN4220 and Newman of human origin and two poultry associated strains (ch24 and ph1; neither of which contained pAvX plasmid), were supplemented with a plasmid carrying a StpC encoding cassette under its native promoter or an empty plasmid. Before supplementation all non-virulent strains exhibited low proteolytic activity as determined using skim milk agar plate assay. In the same assay, the supplemented strains exhibited high level of proteolytic activity, comparable to wild-type StpC producers. The virulence of recombinant strains was compared in the chicken embryo model. No significant difference in the survival rate was observed in any of the tested pairs between a particular strain supplemented with the StpC expressing plasmid or an empty one (Figure 2). These results clearly indicate that StpC expression alone does not determine the virulence level in the chicken embryo model.

### Alpha-haemolysin (HLA) and bifunctional autolysin (ATL) constitute the fingerprint of a virulent proteome

Our further proteomic analysis aimed at finding correlations, if any, between the expression of particular exoproteins and the virulence in the chicken embryo model. Two-channel analysis of DIGE gels identified 263 protein spots characteristic for VIR strains only, within the 12 pairs of compared VIR and NVIR exoproteomes. LC-MS/MS analysis demonstrated that the spots represent 46 different staphylococcal proteins, as multiple spots originated from the same protein, suggesting posttranslational modifications (Table 2 and S3 Table). Of those proteins, only alpha-haemolysin (HLA) and bifunctional autolysin (ATL) were identified in all five analyzed VIR strains. This could indicate that these two proteins constitute a core of a virulent proteome; nevertheless, further analysis demonstrated that the interpretation is more complicated (see below). Formate-tetrahydrofolate ligase (FTHS) and phosphoenolpyruvate carboxykinase (PCKA) were identified in the exoproteomes of all but 1 (CH23) virulent strains. Fifteen other proteins were identified each in two VIR strains and 27 further proteins were characteristic for single analyzed VIR exoproteomes.

**Table 2:** **A list of proteins identified when analyzing the spots differentiating (up regulated) in VIR strains exoproteomes compiled according to the number of strains with identifications within the group of five VIR strains**.

**Protein name**	**Acronym**	**Number of strains with positive identification within the group of five virulent strains**	**Total number of identifications in five virulent strains**	**Strain where the protein was identified**
*Bifunctional autolysin^a^*	*ATL*	*5*	*9*	*CH3, CH5, CH9, CH23, PA2*
Alpha-hemolysin	HLA	5	43	CH3, CH5, CH9, CH23, PA2
*Formate-tetrahydrofolate ligase*	*FTHS*	*4*	*8*	*CH3, CH5, CH9, PA2*
*Phosphoenolpyruvate carboxykinase [ATP]*	*PCKA*	*4*	*10*	*CH3, CH5, CH9, PA2*
*Alcohol dehydrogenase*	*ADH*	*2*	*2*	*CH23, PA2*
*Catalase*	*CATA*	*2*	*2*	*CH5, CH9*
ATP-dependent Clp protease ATP-binding subunit ClpL	CLPL	2	5	CH3, CH5
Enterotoxin type D	ETXD	2	19	CH3, CH5
*Lipase 1*	*LIP1*	*2*	*46*	*CH3, CH5*
*Lipase 2*	*LIP2*	*2*	*47*	*CH3, CH5*
Probable malate:quinone oxidoreductase 2	MQO2	2	2	CH3, CH5
*Thermonuclease*	*NUC*	*2*	*2*	*CH3, CH9*
*1-phosphatidylinositol phosphodiesterase*	*PLC*	*2*	*11*	*CH3, CH5*
*Putative surface protein SAV2496/SAV2497*	*PLS*	*2*	*23*	*CH9, PA2*
Surface protein G	SASG	2	20	CH9, PA2
*Serine-aspartate repeat-containing protein E*	*SDRE*	*2*	*5*	*CH3, CH5*
Transketolase	TKT	2	2	CH3, CH5
*Putative universal stress protein SA1532*	*Y1532*	*2*	*2*	*CH3, CH5*
N-acetylmuramoyl-L-alanine amidase domain-containing protein SAOUHSC_02979	Y2979	2	20	CH3, CH5
*Alkyl hydroperoxide reductase subunit C*	*AHPC*	*1*	*1*	*CH23*
*Fructose-bisphosphate aldolase*	*ALF2*	*1*	*1*	*CH5*
Beta-lactamase	BLAC	1	1	CH5
Diacetyl reductase [(S)-acetoin forming]	BUTA	1	1	CH5
Clumping factor B	CLFB	1	2	CH23
GTP-sensing transcriptional pleiotropic repressor CodY	CODY	1	1	CH5
Dihydrolipoyl dehydrogenase	DLDH	1	1	CH3
Chaperone protein DnaK	DNAK	1	1	CH5
Elongation factor G	EFG	1	1	CH5
*Elongation factor Tu*	*EFTU*	*1*	*1*	*CH5*
*Glyceraldehyde-3-phosphate dehydrogenase 1*	*G3P1*	*1*	*1*	*CH3*
*2,3-bisphosphoglycerate-dependent phosphoglycerate mutase*	*GPMA*	*1*	*1*	*PA2*
Urocanate hydratase	HUTU	1	1	CH5
*Probable transglycosylase isaA*	*ISAA*	*1*	*1*	*CH3*
Ribose-phosphate pyrophosphokinase	KPRS	1	2	CH5
Pyruvate kinase	KPYK	1	2	CH5
Uncharacterized leukocidin-like protein 1	LUKL1	1	1	CH3
Formate acetyltransferase	PFLB	1	2	CH5
Polyribonucleotide nucleotidyltransferase	PNP	1	1	CH5
Immunoglobulin G-binding protein A	SPA	1	1	CH3
Serine protease splA	SPLA2	1	1	CH3
Serine protease splB	SPLB	1	1	CH3
Glutamate–tRNA ligase	SYE	1	1	CH5
Threonine–tRNA ligase	SYT	1	1	CH5
Thioredoxin reductase	TRXB	1	1	CH5
UPF0355 protein MRSA252	UP355	1	1	CH3
UPF0447 protein SAR0593	Y593	1	1	CH5

a *Proteins identified as differentiating in both VIR and NVIR strain proteomes are italicized (see also subchapter “Analysis of non-overlapping spots with identical identification”)*.

One-channel analysis of the exoproteomes clearly demonstrated two major types of patterns in 2-DE. The first type is characterized by a large number of spots, none of which strongly predominates (CH3, CH5, ph1, ch24, pa3). The second type shows a relatively small number of spots with several large spots dominating the image (CH9, CH23, PA2, ph2, ch22) (Figure 1A). When the VIR and NVIR proteomes are compared within the first type, HLA, enterotoxin type D (ETXD), lipase 1 (LIP1), lipase 2 (LIP2), and N-acetylmuramoyl-L-alanine amidase domain-containing protein (Y2979) are found in all VIR strains. Interestingly, these proteins were identified with extraordinary frequency (16; 13; 17; 18; and 7 identifications for CH3, and 18; 6; 30; 28; and 13 for CH5, respectively), as the number of identifications of those five proteins constitutes 74.7 and 69.3% of all protein identifications in the exoproteomes of the CH3 and CH5 strains, respectively. When VIR and NVIR proteomes are compared within the second type, none of the additional proteins characteristic for all VIR strains are identified apart from ATL and HLA mentioned previously. However, for the CH9 and PA2 virulent strains, PCKA, putative surface protein SAV2496 (PLS), and surface protein G (SASG) were identified as upregulated regardless of the non-virulent strain (ch22 or ph2) used for comparison. These proteins also constituted a major part of the exoproteome, 67.7 and 83.3% of all protein identifications in CH9 and PA2, respectively.

### Glutamylendopeptidase expression is a fingerprint of a non-virulent exoproteome

One hundred and seventy six protein spots were detected as positively differentiating in non-virulent exoproteomes within 12 performed comparisons with the virulent counterparts. These spots originated from 66 different proteins, as identified by MS analysis. Glutamylendopeptidase (SSPA, also referred to as V8 protease) expression differentiated all analyzed NVIR proteomes compared to those of virulent strains (Table 3 and S4 Table). Expression of alcohol dehydrogenase (ADH), alkyl hydroperoxide reductase subunit C (AHPC), and enolase (ENO) was detected in four out of the five non-virulent exoproteomes. Expression of seven other proteins was detected in three NVIR strains. Another nine proteins were identified each in two NVIR strains. Finally, 46 proteins were identified each in a single NVIR strain, (Table 3 and S4 Table).

**Table 3:** **A list of proteins identified when analyzing the spots differentiating (up regulated) in NVIR strains exoproteomes compiled according to the number of strains with identifications within the group of five NVIR strains**.

**Protein name**	**Acronym**	**Number of strains with positive identification within the group of five non-virulent strains**	**Total number of identifications in five non-virulent strains**	**Strain where the protein was identified**
Glutamyl endopeptidase	SSPA	5	29	ch22, ch24, pa3, ph1, ph2
*Alcohol dehydrogenase^a^*	*ADH*	*4*	*4*	*ch24, pa3, ph1, ph2*
*Alkyl hydroperoxide reductase subunit C*	*AHPC*	*4*	*10*	*ch22, ch24, pa3, ph2*
Enolase	ENO	4	9	ch24, pa3, ph1, ph2
*Bifunctional autolysin*	*ATL*	*3*	*4*	*ch24, pa3, ph2*
*Catalase*	*CATA*	*3*	*4*	*pa3, ph1, ph2*
*Glyceraldehyde-3-phosphate dehydrogenase 1*	*G3P1*	*3*	*4*	*ch22, pa3, ph2*
*Phosphoenolpyruvate carboxykinase [ATP]*	*PCKA*	*3*	*11*	*ch24, pa3, ph1*
*1-phosphatidylinositol phosphodiesterase*	*PLC*	*3*	*11*	*ch24, pa3, ph1*
Staphopain B	SSPB	3	14	ch24, pa3, ph2
*Lipase 1*	*LIP1*	*2*	*11*	*ch24, pa3*
Collagen adhesin	CNA	2	8	ch24, ph1
3-hydroxyacyl-[acyl-carrier-protein] dehydratase FabZ	FABZ	2	2	ch24, ph1
*Formate–tetrahydrofolate ligase*	*FTHS*	*3*	*8*	*pa3, ph1, ph2*
Molecular chaperone Hsp31 and glyoxalase 3	HCHA	2	2	ch24, ph2
*Probable transglycosylase IsaA*	*ISAA*	*2*	*2*	*ch24, pa3*
Pyruvate dehydrogenase E1 component subunit beta	ODPB	2	4	ch22, ch24
Phosphoglycerate kinase	PGK	2	4	pa3, ph2
*Serine-aspartate repeat-containing protein E*	*SDRE*	*2*	*11*	*ch22, ph2*
Staphopain A	SSPP	2	2	pa3, ph1
Acetate kinase	ACKA	1	1	ch24
Fructose-bisphosphate aldolase class 1	ALF1	1	1	ch24
*Fructose-bisphosphate aldolase*	*ALF2*	*1*	*1*	*ch24*
Catalase-like protein	CATB	1	1	ph2
10 kDa chaperonin	CH10	1	1	ch24
Clumping factor A	CLFA	1	1	ph1
Cysteine synthase	CYSK	1	4	ch24
Deoxyribose-phosphate aldolase 2	DEOC2	1	1	pa3
Alanine dehydrogenase 2	DHA2	1	4	ch24
Elongation factor Ts	EFTS	1	1	ch24
*Elongation factor Tu*	*EFTU*	*1*	*2*	*ch24*
Enterotoxin type H	ETXH	1	2	ch24
Glucose-6-phosphate isomerase	G6PI	1	1	ch24
Phosphoribosyl aminoimidazole carboxylase, catalytic subunit	gi|87162294	1	1	ph1
*2,3-bisphosphoglycerate-dependent phosphoglycerate mutase*	*GPMA*	*1*	*1*	*pa3*
Gamma-hemolysin component A	HLGA	1	1	ch24
3-hexulose-6-phosphate synthase	HPS	1	1	ch24
Inosine-5′-monophosphate dehydrogenase	IMDH	1	2	ch24
6-phosphofructokinase	K6PF	1	2	ch24
L-lactate dehydrogenase 1	LDH1	1	1	ch24
*Lipase 2*	*LIP2*	*1*	*4*	*ch22*
Lipoteichoic acid synthase	LTAS	1	1	ch22
Molybdenum cofactor biosynthesis protein B	MOAB	1	1	ph1
*Thermonuclease*	*NUC*	*1*	*1*	*pa3*
Ornithine aminotransferase 2	OAT2	1	1	pa3
Pyruvate dehydrogenase E1 component subunit alpha	ODPA	1	2	ch22
*Putative surface protein SAV2496/SAV2497*	*PLS*	*1*	*1*	*ch24*
Putative peptidyl-prolyl cis-trans isomerase	PPI1	1	1	pa3
Phosphate acetyltransferase	PTAS	1	1	pa3
Phosphocarrier protein HPr	PTHP	1	2	ph1
50S ribosomal protein L14	RL14	1	1	pa3
50S ribosomal protein L5	RL5	1	1	pa3
30S ribosomal protein S8	RS8	1	2	pa3
Superoxide dismutase [Mn/Fe] 1	SODM1	1	2	ch24
Prophage-derived single-stranded DNA-binding protein	SSBP	1	1	ph1
Phenylalanine–tRNA ligase alpha subunit	SYFA	1	2	ch24
Signal transduction protein TRAP	TRAP	1	1	ph1
Uracil phosphoribosyltransferase	UPP	1	1	ch24
Uncharacterized N-acetyltransferase SA1019	Y1019	1	1	ch24
*Putative universal stress protein SA1532*	*Y1532*	*1*	*2*	*pa3*
UPF0173 metal-dependent hydrolase SAB1566c	Y1566	1	1	ch24
Uncharacterized protein SA1692	Y1692	1	1	ch24
Uncharacterized oxidoreductase SAS2370	Y2370	1	1	ch24
Uncharacterized protein SA0829	Y829	1	1	ch24
UPF0477 protein SA0873	Y873	1	1	ch24
Uncharacterized protein SAOUHSC_00997	Y997	1	3	ch24

a *Proteins identified as differentiating in both VIR and NVIR strain proteomes are italicized (see also subchapter “Analysis of non-overlapping spots with identical identification”)*.

Analogically to the proteins characteristic for VIR exoproteomes described in the previous chapter, those characteristic for NVIR exoproteomes were also identified in multiple protein spots. This phenomenon was most pronounced for PCKA, PLC, LIP1, and cysteine protease staphopain B (SSPB) (each identified in at least ten different spots in the analyzed exoproteomes). Moreover, serine-aspartate repeat-containing protein E (SDRE) was identified within nine different spots in strain ch22 exoproteome compared with the CH9, CH23, and PA3 virulent strains.

**Figure 3:**
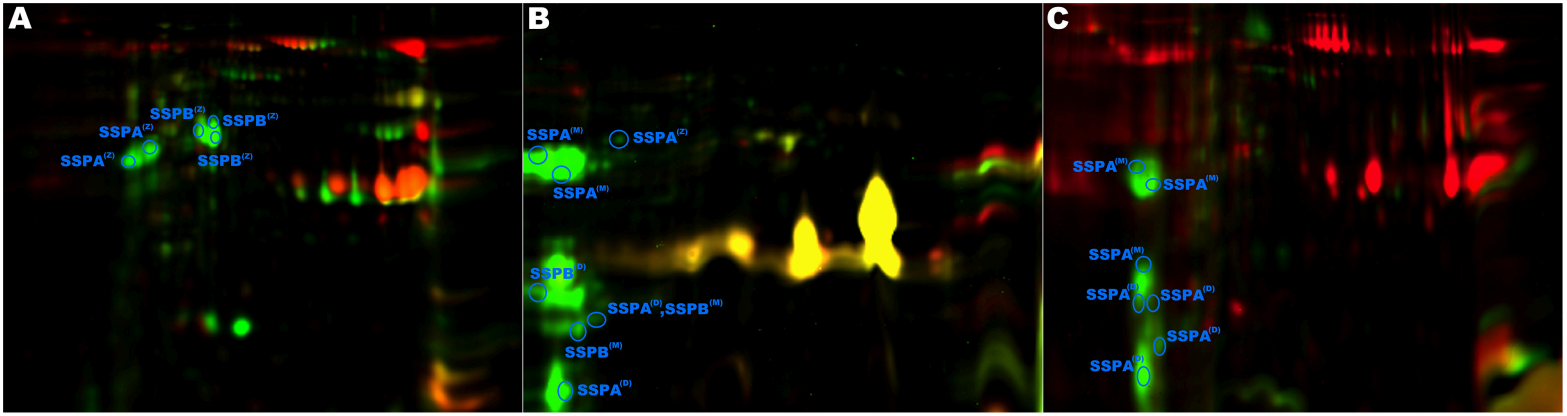
**Analysis of glutamylendopeptidase (SSPA) and staphopain B (SSPB) posttranslational processing**. SSPA and SSPB were identified only in the non-virulent strains. **(A)** Strain pa3 contained only zymogen (Z in the superscript) forms of both proteases (as evidenced by approximate molecular weight and tryptic peptide coverage), indicating a disrupted activation pathway. Strains ph2 **(B)** and ph1 **(C)** contained the zymogen, the mature (M in the superscript) forms, and the degradation (D in the superscript) products of the proteases.

Glutamylendopeptidase (SSPA) was previously suggested to play a role in virulence of *S. aureus* (Coulter et al., 1998; Kolar et al., 2013). We therefore found it surprising that this enzyme was identified only in the exoproteomes of NVIR but not VIR strains, especially that the enzyme was abundantly expressed (29 spots in all analyzed exoproteomes originated from SSPA). It was demonstrated previously that SSPA is produced as a zymogen activated by a metalloprotease aureolysin. We therefore hypothesized that possibly it is not SSPA itself, but a deficiency in its activation that is characteristic for NVIR strains, especially that only mature SSPA activates SSPB zymogen which in turn was also suggested of importance in staphylococcal virulence (Rice et al., 2001). By analyzing sequence coverage in LC-MS/MS identifications and approximate molecular weights in 2-DE, we were able to distinguish, with relatively high confidence, spots belonging to the zymogen and the mature protease (S1 Figure). Seven SSPA-originating spots contained tryptic peptides encompassing a part of a profragment or an intact Asn68-Val69 zymogen processing site (Nickerson et al., 2007), directly demonstrating that these spots originated from the SSPA zymogen. In the remaining 22 spots originating from SSPA, none of the tryptic peptides corresponded to the profragment. The approximate molecular weight of proteins contained within most of those spots indicates that they originate form mature SSPA, therefore SSPA activation is not affected in these strains. The molecular weight of certain spots corresponded to the zymogen and here the identification was ambiguous. Moreover, several spots with approximate molecular weight below that expected for mature SSPA were found, in which the peptide coverage suggested processing at the C-terminus. Overall, the exoproteomes of strains ch22, ch24, and ph2 contained both the zymogen and the mature form of SSPA. Exclusively in ph1, only the mature form was detected. Interestingly, pa3 contained only SSPA zymogen, which may suggest impaired activation in this strain (Figure 3 and S1 Figure). The impaired activation cascade in pa3 is further supported by the fact that SSPB is also found in its zymogen form in this strain, whereas in other strains mature SSPA correlates with mature SSPB, as expected according to the activation mechanism described previously (Nickerson et al., 2007).

### Analysis of non-overlapping spots with identical identification

**Figure 4:**
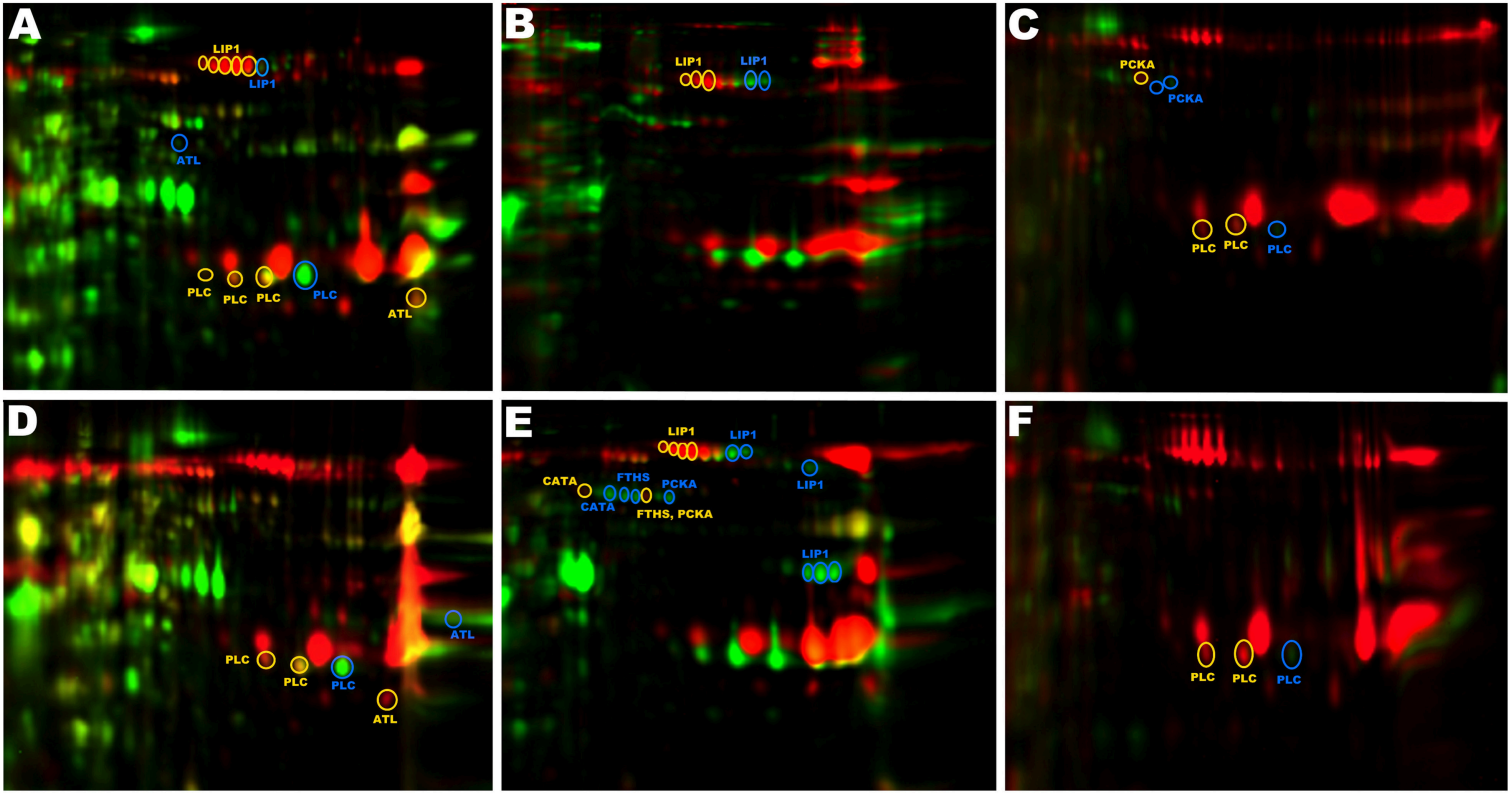
**Non-overlapping spots with identical identifications**. Spots originating from the same protein do not overlap on 2D gels due to genetic variation and differential posttranslational processing. **(A)** CH3/ch24, **(B)** CH3/pa3, **(C)** CH3/ph1, **(D)** CH5/ch24, **(E)** CH5/pa3, **(F)** CH5/ph1. Red (yellow circles)—VIR strain; green (blue circles)—NVIR strain; orange (yellow circles)—overlapping spots.

2D-DIGE analysis is best suited for detecting relatively small differences between closely related proteomes. In this study, exoproteomes of distantly related strains were compared while 2D-DIGE was successfully used as a convenient means of eliminating proteins of housekeeping function or expressed regardless of the strain virulence (spots yellow and orange in color) from further analysis. To this end, only differentiating spots were subject to further analysis (red and green spots, reflecting proteins with higher abundance in VIR and NVIR proteomes, respectively). Interestingly, however, identical proteins were often detected in multiple spots, both within a single exoproteome (multiple spots with identical identification), but also in differential analysis of two proteomes (non-overlapping spots with identical identification; italicized in Tables 2, 3). Such differences may reflect genetic variation or posttranslational modifications or both and may have an important impact on protein function. As such, the sources of the observed variability were analyzed in greater detail for LIP1, PLC, FTHS, PCKA, CATA, and ATL, since the highest heterogeneity was observed among those proteins (Figure 4 and S2 Figure).

Spots originating from LIP1 derived from different strains do not overlap because of the differences in pI (Figures 4A,B,E). Sequence coverage in LC-MS/MS identification demonstrates that the analyzed spots originate from the LIP1 precursor. The LIP1 precursor derived from the NVIR strains (ch24 and pa3) is shifted toward higher pI relative to the LIP1 derived from the VIR strains (CH3 and CH5). To evaluate if genetic differences or posttranslational modifications are responsible, we compared the open reading frames encoding LIP1 obtained from shotgun sequencing of respective strains. The amino acid sequences of LIP1 derived from the VIR strains (calculated pI = 6.21) was identical to that derived from *S. aureus* Mu50 (ATCC 700699), chosen as the best reference sequence by the software for protein identification. In turn, the sequences derived from the NVIR strains differed by 9 and 7 amino acids, respectively, for strains ch24 and pa3 (calculated pI = 6.49 and 6.31, respectively). Therefore, the partial non-overlap of spots originating from LIP1 is explained by differences in the amino acid sequence of the protein derived from different strains. The exoproteome of pa3 contains additional spots originating form LIP1, but located in a different part of the gel. Here, however, the approximate molecular weight and sequence coverage clearly demonstrate that these spots originate form intermediate and mature forms of LIP1.

Similarly to LIP1, allelic variants are responsible for shifting of the spots originating from PLC from the NVIR strains (ch24 and ph1) toward higher pI, compared to those originating from the VIR strains (CH3 and CH5) (Figures 4A,C,D,F). The same is true for FTHS, although here the protein originating from the NVIR strain (pa3) is shifted toward lower pI compared to that derived from the VIR strain (CH5). In addition, FTHS is more abundantly expressed in pa3, compared to CH5 (Figure 4E). Similarly, in the case of PCKA (Figures 4C,E), the protein sequence and corresponding pI differs in the VIR and NVIR strains (calculated pI of 5,74 and 5,89, respectively). In parallel, proteolytic processing at the C-terminus of PCKA is observed in strain ph1 (Figure 4C), as evidenced by the slight shift to lower molecular weight and the peptide coverage obtained. Proteolytic processing at the C-terminus is also observed within CATA in the NVIR strain (pa3) but not in CH5 (Figure 4E), which in this case is solely responsible for the lack of co-localization of corresponding spots. Overall, this analysis concludes in an interesting observation that the exoproteomes of the VIR an NVIR strains not only differ in the protein content, but are also characterized by distinct genetic variants and differences in posttranslational processing. These phenomena may constitute yet another level of complexity necessary to uncover in order to better understand the role and interplay of virulence determinants; however, our limited sample does not allow establishing meaningful correlations.

When spots differentiating the VIR from NVIR strains were analyzed, bifunctional autolysin (ATL) was identified in all VIR exoproteomes. At the same time, analysis of spots differentiating the NVIR strains also resulted in identification of ATL. In this case, the difference in spot localization on 2D-gels was not related to genetic differences or simple processing. ATL is a large protein exhibiting two enzymatic activities. The N-terminal domain exhibits N-acetylmuramoyl-L-alanine amidase activity, whereas the C-terminal part is an endo-beta-N-acetylglucosaminidase (Gotz et al., 2014). The tryptic peptides identified in ATL containing spots originating from the VIR strains aligned almost exclusively (8/9) with the N-terminal part of ATL (amidase), while those from NVIR strains mostly (3/4) to the C-terminal part (glucosaminidase). This suggests that the amidase activity of ATL may be important in virulence; however, the mechanism determining differential stability of the two domains of a single protein and the role of the ATL amidase domain in pathogenesis, if any, remain unknown.

## Discussion

We demonstrated previously that, among poultry-derived *S. aureus* strains, those exhibiting high virulence in a chicken embryo model are genetically distinct from non-virulent strains (Polakowska et al., 2012). In this study, we applied proteomics to elucidate how genetic differences are reflected in the extracellular proteome and to identify proteomic signatures of virulent and non-virulent strains. Especially the latter were notoriously neglected in previous analyses, while the concept of “virulence attenuators” gains importance in understanding of commensal coexistence of opportunistic pathogens (Merhej et al., 2013).

The exoproteomes of five VIR and five NVIR strains were analyzed by 2D-electrophoresis demonstrating two major exoprotein patterns which, however, did not follow the VIR/NVIR division. The exoproteomes were either dominated by the presence of StpC and contained relatively few other spots (CH9, CH23, PA2, ph2, and ch22) or contained a large number of spots without any dominant ones (remaining strains). Interestingly, StpC was previously postulated as an avian host-specific virulence factor (Lowder et al., 2009), which contradicts our findings. To further investigate this issue, we tested the effect of StpC supplementation on the virulence of a non-virulent, StpC-negative strains. We found that StpC supplementation was not sufficient to increase the virulence of the non-virulent strains, demonstrating that StpC alone does not significantly affect the virulence potential. However, these findings do not exclude the role of StpC in poultry-host preference.

We compared matched pairs of VIR and NVIR exoproteomes within the two groups defined above. Collectively, in these analyses, the number of spots characteristic for the VIR strains was higher than the number of those specific for NVIR strains. Interestingly, however, the spots characteristic for the VIR strains originated from a smaller number of individual proteins than those specific for the NVIR strains, which indicates significant posttranslational modification of proteins within the VIR strains. The identification of multiple spots within the VIR exoproteomes as originating from a single protein also indicates their high expression. On the other hand, the NVIR exoproteomes are more complex (contain a larger number of different proteins), indirectly suggesting that commensal colonization possibly requires finer tune up compared to brute force pathogenesis. The last conclusion is however highly speculative.

The analyzed exoproteomes were very heterogeneous. For example, the exoproteome of the virulent strain CH23 differed only in five proteins from the exoproteome of the non-virulent strain ch22, whereas in another pair (CH5/ch24) 42 differentiating proteins were identified. Significant heterogeneity within the exoproteomes was, however, expected, given the high plasticity of staphylococcal genomes, and was experimentally documented previously (Ziebandt et al., 2010), while we were interested in identifying virulence patterns/markers within this heterogeneity. Comparison of the VIR and NVIR proteomes identified HLA as a possible marker of the former group. HLA (haemolysin alpha; alpha toxin) is a pore forming toxin penetrating host cell membranes and resulting in their lysis. Studies in animal models of staphylococcal keratitis (McCormick et al., 2009) and pneumonia (Bubeck Wardenburg et al., 2007) document an important role of HLA in virulence, nevertheless its role in avian infection models has not been directly tested. It is known, however, that HLA uses ADAM 10 as a receptor and HLA-driven toxicity depends on the amount of this receptor which differs among cell types and/or the source species (Berube and Bubeck Wardenburg, 2013). High expression of ADAM 10 documented in many tissues of the chicken embryo (Hall and Erickson, 2003), including epidermis during formation of feather buds (Lin et al., 2011) indirectly suggests possible involvement of HLA in staphylococcal virulence in this specie, however direct evidence is missing. Secretion of HLA in *in vitro* cultures characterizes particular clonal types of *S. aureus*, among other, lineage CC5 (Monecke et al., 2014). This is consistent with our findings since the virulent strains tested in our study belonged to lineage CC5 (Polakowska et al., 2012). Due to the limited number of data, the production of HLA could not have been correlated with the host species (Monecke et al., 2014). However, the secretion of HLA only by the poultry-derived strains exhibiting high virulence in the chicken embryo model, as evidenced in our study, may suggest the toxin as a host-associated virulence factor; nevertheless, this is purely speculative and requires further testing.

The N-terminal domain of the major staphylococcal autolysin (ATL) was the second identified protein characteristic for all VIR strains, but not found in the NVIR exoproteomes. Interestingly, the non-virulent strains contained either the C-terminal domain of ATL (ch24 and ph2) or no ATL at all. Only in the NVIR pa3 exoproteome, the N-terminal domain and the middle part of ATL have been identified. ATL is a cell-wall associated bifunctional enzyme involved in daughter cell separation (Sugai et al., 1995; Takahashi et al., 2002). Neither the direct toxicity of ATL toward chicken cells nor the potential indirect involvement of ATL in virulence of *S. aureus* has not been investigated, according to our knowledge. It was demonstrated, however, that in closely related species, *S. epidermidis* and *S. lugdunensis, atl*-null strains are attenuated in an intravascular catheter–associated infection rat model (Rupp et al., 2001) and in a *Caenorhabditis elegans* model, respectively (Gibert et al., 2014). It is unclear, however, whether the decreased virulence was associated with an overall decreased fitness or reflected some specific function of the autolysin. ATL was also implicated in biofilm formation (Bose et al., 2012), which has an established role in staphylococcal virulence. Interestingly, we found that SSPA was expressed exclusively by the NVIR strains, while this protease has been reported to degrade ATL thus exerting negative effect on ATL mediated biofilm formation in *S. aureus* (Chen et al., 2013). Our data suggests SSPA mediated degradation of the N-terminal part of ATL only since the N-terminal part of ATL was found exclusively in VIR proteomes where SSPA was not present and in the pa3 exoproteome, where SSPA was identified only in the form of an inactive zymogen.

The initial translation product of the *atl* gene undergoes proteolytic processing that yields two catalytically active proteins: an amidase (AM) and a glucosaminidase (GL), which corroborates the findings of this study. However, limited data is available to distinguish the roles of these two enzymatic activities in staphylococcal physiology. It has only been demonstrated that, in parallel to the enzymatic activity, AM exhibits adhesion-like vitronectin-binding activity (Heilmann et al., 1997), while GL has a DNA-binding capacity (Grilo et al., 2014). The limited amount of information does not allow us to speculate on the potential role of differential stability of AM and GL in the VIR and NVIR strains documented in this study. Nevertheless, it is worth noting that not only the expression of ATL-derived amidase was characteristic for the VIR strains. Certain VIR strains (CH3 and CH5) were also characterized by expression of Y2979 protein, which contains an N-acetylmuramoyl-L-alanine amidase domain similar to AM. Moreover, Y2979 secretion is affected by mutations in accessory secretory systems responsible for the export of staphylococcal virulence factors (Siboo et al., 2008; Sibbald et al., 2010). However, the significance of this fact remains unknown.

Besides the above-described functions, ATL was implicated in excretion of cytoplasmic proteins. A study by Pasztor et al. has shown that mutation within the *atl* gene affected excretion of 22 typically cytoplasmic proteins. Interestingly, the ATL related excretion was discriminatory since the most abundant cytoplasmic proteins were not found outside the cell (Pasztor et al., 2010). In our study, more cytoplasmic proteins were identified in the non-virulent exoproteome (48), compared to that of the virulent strains (28). The correlation of this fact and differential ATL stability in the VIR and NVIR strains is unknown. However, since excretion of cytosolic proteins is clearly connected with bacterial virulence (Gotz et al., 2015), the imbalance in such proteins in the exoproteomes of the VIR and NVIR strains opens a field for further research. In summary, despite data linking the presence or activity of ATL with staphylococcal phenotype its role in virulence is questionable.

Recent findings indicate that the virulence level is related not only to the expression of virulence factors but also anti-virulence genes, which are characteristic for nonpathogenic strains of otherwise pathogenic bacteria (Merhej et al., 2013). While analyzing the protein spots characteristic for NVIR proteomes only, we identified SSPA expression as a fingerprint of the NVIR exoproteome. This fact was at first unexpected, since staphylococcal proteases have been widely implicated in virulence. In particular, a number of reports consistently suggested the role of SSPA in staphylococcal virulence based on certain *in vitro* properties and indirect observations (Prokešová et al., 1988, 1992; Karlsson et al., 2001) However, the data from *in vivo* models is relatively inconclusive (Coulter et al., 1998; Rice et al., 2001), clearly indicating only that although a single protease gene knock-out may not change the virulence, the orchestrated action of multiple secreted staphylococcal proteases may have an profound effect on the growth and survival of *S. aureus* in the infected host (Kolar et al., 2013). Therefore, advanced combination knock-outs are needed to unambiguously indicate the role of particular proteases in staphylococcal virulence. Moreover, all the above reports considered virulence in human/mammalian models, while Lowder and colleagues (Lowder et al., 2009) observed *sspA* pseudogenization in some poultry isolates, which indirectly suggests dispensability of SSPA in the avian host. Although the avian strains characterized in our study all contained the intact *sspA* gene, our data indirectly suggest the dispensability of SSPA in staphylococcal virulence in an avian host since its expression was not identified in any of the VIR strains analyzed.

SSPA is coexpressed with SSPB within a single operon (Rzychon et al., 2003). Consistently, SSPB expression was found exclusively in the NVIR strains (ch24, pa3, and ph2). Previous studies identified SSPB as an important staphylococcal virulence factor (Potempa and Pike, 2009; Smagur et al., 2009; Ohbayashi et al., 2011), but as for SSPA, the studies involved mammalian models only. Our data indicates that SSPB is dispensable for *S. aureus* virulence in the avian host since its expression was not identified in any of the virulent strains.

Besides the differential expression of multiple proteins, genetic variability (allelic variants) further contributes to the heterogeneity of staphylococcal proteomes as exemplified here by LIP1, PLC, and FTHS. This strengthens the importance of combining genomic and proteomic approaches to exoproteome analysis. The extent and impact of allelic variability on the virulence potential of staphylococci remains almost completely unexplored and is certainly worth further insight.

Post-translational modifications provide yet another source of variability within the exoproteomes. Secreted proteases are important agents modulating the extracellular as well as cell-wall-bound proteome, which has important implications in staphylococcal virulence (Kantyka et al., 2013; Kolar et al., 2013). This study corroborates the previous findings on zymogen maturation, but at the same time provides evidence that proteolytic processing is more extended and complex than simple zymogen activation. This is exemplified by the differential stability of the ATL domains analyzed here in some detail, but also by the fact that the extent of zymogen processing is dissimilar in different strains, certain proteins are processed at sites other than the classical activation site, and proteins such as CATA and PCKA are processed to smaller stable fragments than what is classically considered as their mature form. Besides zymogen activation, the impact on those processes on staphylococcal physiology remains to be uncovered.

Yet another level of variability within the exoproteomes is provided by posttranslational modifications other than proteolysis. These are manifested as populations of spots originating from a single protein and having identical molecular weight but differing in pI. These modifications were observed in this and many other studies, but were not analyzed further and their nature remains unknown, though certainly worth further investigation.

Summarizing, despite the fact that the extracellular proteomes of the VIR and NVIR strains are heterogeneous within each group, we identified fingerprint proteins characteristic for each phenotype. Haemolysin alpha (HLA) is expressed exclusively in virulent strains and hence may be considered as a poultry-host-associated virulence factor. Serine protease SSPA is characteristic for the exoproteomes of non-virulent strains only. It was demonstrated here that staphopain C, a protease previously implicated in staphylococcal virulence in the avian host, is alone not capable of increasing the pathogenicity of non-virulent avian strains and is dispensable in the virulent phenotype. Further, this study demonstrates that besides expression levels of different proteins, posttranslational modifications and allelic variability significantly contribute to the changeability within staphylococcal extracellular proteomes. The latter processes are largely unexplored but clearly merit in-depth characterization.

## Author contributions

EB and BW designed the study. EB, IW, UJ, SK, MB, KP, ML, MK, and AS performed the experiments. EB, SK, KP, GD, AS, AF, JM, AD, and BW analyzed and interpreted data. EB and BW wrote the manuscript. All authors revised the manuscript and agreed to be accountable for all aspect of the work herein.

## Funding

This research was supported by funds granted by the National Science Centre (NCN, Poland) on the basis of the decision no. DEC-2012/07/D/NZ2/04282 (to BW). Proteomics were carried out with the equipment purchased through European Union structural funds, grant POIG.02.01.00-12-167/08 (Malopolska Centre of Biotechnology).

### Conflict of interest statement

The authors declare that the research was conducted in the absence of any commercial or financial relationships that could be construed as a potential conflict of interest.
