# Supplementary material for: Identification of Secreted Exoproteome Fingerprints of Highly-Virulent and Non-Virulent Staphylococcus aureus Strains
Source: Front Cell Infect Microbiol. 2016 May 6;6:51. doi: 10.3389/fcimb.2016.00051 (PMC4874363; doi:10.3389/fcimb.2016.00051)
Supplement: Supplementary file 1 [file DataSheet1.DOC]

**Supporting information for**

**Identification of secreted exoproteome fingerprints of highly-virulent and non-virulent *Staphylococcus aureus* strains**

**Emilia Bonar^1^, Iwona Wojcik^1^, Urszula Jankowska^2^, Sylwia Kedracka-Krok^2,3^, Michal Bukowski^1^, Klaudia Polakowska^4^, Marcin W. Lis^5^, Maja Kosecka-Strojek^4^, Artur J. Sabat^6^, Grzegorz Dubin^2,4^, Alexander W. Friedrich^6^, Jacek Miedzobrodzki^4^, Adam Dubin^1,2^, Benedykt Wladyka^1*^**

**S1 Table. A list of proteins differentiating in pairs VIR/NVIR strain.**

| **CH3/ch24** | | |
| --- | --- | --- |
| Acronym | Protein name | Number of identifications |
| ATL | Bifunctional autolysin | 1 |
| CLPL | ATP-dependent Clp protease ATP-binding subunit ClpL | 1 |
| ETXD | Enterotoxin type D | 4 |
| HLA | Alpha-hemolysin | 8 |
| ISAA | Probable transglycosylase isaA | 1 |
| LIP1 | Lipase 1 | 7 |
| LIP2 | Lipase 2 | 5 |
| NUC | Thermonuclease | 1 |
| PLC | 1-phosphatidylinositol phosphodiesterase | 3 |
| SDRE | Serine-aspartate repeat-containing protein E | 1 |
| SPA | Immunoglobulin G-binding protein A | 1 |
| SPLA2 | Serine protease splA | 1 |
| SPLB | Serine protease splB | 1 |
| TKT | Transketolase | 1 |
| Y2979 | N-acetylmuramoyl-L-alanine amidase domain-containing protein SAOUHSC_02979 | 2 |
| **ch24/CH3** | | |
| AHPC | Alkyl hydroperoxide reductase subunit C | 2 |
| ATL | Bifunctional autolysin | 1 |
| CH10 | 10 kDa chaperonin | 1 |
| CNA | Collagen adhesin | 3 |
| CYSK | Cysteine synthase | 2 |
| DHA2 | Alanine dehydrogenase 2 | 1 |
| EFTU | Elongation factor Tu | 2 |
| ETXH | Enterotoxin type H | 1 |
| HLGA | Gamma-hemolysin component A | 1 |
| IMDH | Inosine-5'-monophosphate dehydrogenase | 1 |
| LIP1 | Lipase 1 | 2 |
| PCKA | Phosphoenolpyruvate carboxykinase [ATP] | 2 |
| PLC | 1-phosphatidylinositol phosphodiesterase | 1 |
| SODM1 | Superoxide dismutase [Mn/Fe] 1 | 1 |
| SSPB | Staphopain B | 2 |
| UPP | Uracil phosphoribosyltransferase | 1 |
| Y1019 | Uncharacterized N-acetyltransferase SA1019 | 1 |
| Y1566 | UPF0173 metal-dependent hydrolase SAB1566c | 1 |
| Y2370 | Uncharacterized oxidoreductase SAS2370 | 1 |
| Y997 | Uncharacterized protein SAOUHSC_00997 | 1 |
|  | | |
| **CH3/pa3** | | |
| ATL | Bifunctional autolysin | 1 |
| ETXD | Enterotoxin type D | 5 |
| HLA | Alpha-hemolysin | 3 |
| LIP1 | Lipase 1 | 3 |
| LIP2 | Lipase 2 | 3 |
| LUKL1 | Uncharacterized leukocidin-like protein 1 | 1 |
| **pa3/CH3** | | |
| ENO | Enolase | 1 |
| FTHS | Formate-tetrahydrofolate ligase | 1 |
| ISAA | Probable transglycosylase IsaA | 1 |
| LIP1 | Lipase 1 | 2 |
| NUC | Thermonuclease | 1 |
| PCKA | Phosphoenolpyruvate carboxykinase [ATP] | 2 |
| PLC | 1-phosphatidylinositol phosphodiesterase | 3 |
| PPI1 | Putative peptidyl-prolyl cis-trans isomerase | 1 |
| RL5 | 50S ribosomal protein L5 | 1 |
| SSPP | Staphopain A | 1 |
| Y1532 | Putative universal stress protein SA1532 | 2 |
|  | | |
| **CH3/ph1** | | |
| DLDH | Dihydrolipoyl dehydrogenase | 1 |
| ETXD | Enterotoxin type D | 4 |
| FTHS | Formate-tetrahydrofolate ligase | 1 |
| G3P1 | Glyceraldehyde-3-phosphate dehydrogenase 1 | 1 |
| HLA | Alpha-hemolysin | 5 |
| LIP1 | Lipase 1 | 6 |
| LIP2 | Lipase 2 | 11 |
| MQO2 | Probable malate:quinone oxidoreductase 2 | 1 |
| PCKA | Phosphoenolpyruvate carboxykinase [ATP] | 1 |
| PLC | 1-phosphatidylinositol phosphodiesterase | 3 |
| UP355 | UPF0355 protein MRSA252 | 1 |
| Y1532 | Putative universal stress protein SA1532 | 1 |
| Y2979 | N-acetylmuramoyl-L-alanine amidase domain-containing protein SAOUHSC_02979 | 5 |
| **ph1/CH3** | | |
| CLFA | Clumping factor A | 1 |
| CNA | Collagen adhesin | 1 |
| ENO | Enolase | 1 |
| FABZ | 3-hydroxyacyl-[acyl-carrier-protein] dehydratase FabZ | 1 |
| gi|87162294 | Phosphoribosylaminoimidazole carboxylase, catalytic subunit | 1 |
| MOAB | Molybdenum cofactor biosynthesis protein B | 1 |
| PCKA | Phosphoenolpyruvate carboxykinase [ATP] | 2 |
| PLC | 1-phosphatidylinositol phosphodiesterase | 1 |
| SSBP | Prophage-derived single-stranded DNA-binding protein | 1 |
| SSPA | Glutamyl endopeptidase | 2 |
| SSPP | Staphopain A | 1 |
| TRAP | Signal transduction protein TRAP | 1 |
|  | | |
| **CH5/ch24** | | |
| ATL | Bifunctional autolysin | 1 |
| BLAC | Beta-lactamase | 1 |
| CLPL | ATP-dependent Clp protease ATP-binding subunit ClpL | 2 |
| EFTU | Elongation factor Tu | 1 |
| ETXD | Enterotoxin type D | 2 |
| HLA | Alpha-hemolysin | 5 |
| KPRS | Ribose-phosphate pyrophosphokinase | 1 |
| LIP1 | Lipase 1 | 6 |
| LIP2 | Lipase 2 | 4 |
| PLC | 1-phosphatidylinositol phosphodiesterase | 2 |
| SDRE | Serine-aspartate repeat-containing protein E | 1 |
| Y2979 | N-acetylmuramoyl-L-alanine amidase domain-containing protein SAOUHSC_02979 | 3 |
| **ch24/CH5** | | |
| ACKA | Acetate kinase | 1 |
| ADH | Alcohol dehydrogenase | 1 |
| AHPC | Alkyl hydroperoxide reductase subunit C | 3 |
| ALF1 | Fructose-bisphosphate aldolase class 1 | 1 |
| ALF2 | Fructose-bisphosphate aldolase | 1 |
| ATL | Bifunctional autolysin | 1 |
| CNA | Collagen adhesin | 4 |
| CYSK | Cysteine synthase | 2 |
| DHA2 | Alanine dehydrogenase 2 | 3 |
| EFTS | Elongation factor Ts | 1 |
| ENO | Enolase | 1 |
| ETXH | Enterotoxin type H | 1 |
| FABZ | 3-hydroxyacyl-[acyl-carrier-protein] dehydratase FabZ | 1 |
| G6PI | Glucose-6-phosphate isomerase | 1 |
| HCHA | Molecular chaperone Hsp31 and glyoxalase 3 | 1 |
| HPS | 3-hexulose-6-phosphate synthase | 1 |
| IMDH | Inosine-5'-monophosphate dehydrogenase | 1 |
| ISAA | Probable transglycosylase IsaA | 1 |
| K6PF | 6-phosphofructokinase | 2 |
| LDH1 | L-lactate dehydrogenase 1 | 1 |
| LIP1 | Lipase 1 | 1 |
| ODPB | Pyruvate dehydrogenase E1 component subunit beta | 2 |
| PCKA | Phosphoenolpyruvate carboxykinase [ATP] | 2 |
| PLC | 1-phosphatidylinositol phosphodiesterase | 1 |
| PLS | Putative surface protein SAV2496/SAV2497 | 1 |
| SODM1 | Superoxide dismutase [Mn/Fe] 1 | 1 |
| SSPA | Glutamyl endopeptidase | 3 |
| SSPB | Staphopain B | 3 |
| SYFA | Phenylalanine-tRNA ligase alpha subunit | 2 |
| Y1692 | Uncharacterized protein SA1692 | 1 |
| Y829 | Uncharacterized protein SA0829 | 1 |
| Y873 | UPF0477 protein SA0873 | 1 |
| Y997 | Uncharacterized protein SAOUHSC_00997 | 2 |
|  | | |
| **CH5/pa3** | | |
| ALF2 | Fructose-bisphosphate aldolase | 1 |
| BUTA | Diacetyl reductase [(S)-acetoin forming] | 1 |
| CATA | Catalase | 1 |
| CLPL | ATP-dependent Clp protease ATP-binding subunit ClpL | 2 |
| DNAK | Chaperone protein DnaK | 1 |
| EFG | Elongation factor G | 1 |
| ETXD | Enterotoxin type D | 2 |
| FTHS | Formate-tetrahydrofolate ligase | 1 |
| HLA | Alpha-hemolysin | 7 |
| HUTU | Urocanate hydratase | 1 |
| KPRS | Ribose-phosphate pyrophosphokinase | 1 |
| KPYK | Pyruvate kinase | 2 |
| LIP1 | Lipase 1 | 12 |
| LIP2 | Lipase 2 | 14 |
| PCKA | Phosphoenolpyruvate carboxykinase [ATP] | 1 |
| PFLB | Formate acetyltransferase | 2 |
| PNP | Polyribonucleotide nucleotidyltransferase | 1 |
| SDRE | Serine-aspartate repeat-containing protein E | 3 |
| SYE | Glutamate-tRNA ligase | 1 |
| SYT | Threonine-tRNA ligase | 1 |
| TRXB | Thioredoxin reductase | 1 |
| Y1532 | Putative universal stress protein SA1532 | 1 |
| Y2979 | N-acetylmuramoyl-L-alanine amidase domain-containing protein SAOUHSC_02979 | 4 |
| Y593 | UPF0447 protein SAR0593 | 1 |
| **pa3/CH5** | | |
| ADH | Alcohol dehydrogenase | 1 |
| AHPC | Alkyl hydroperoxide reductase subunit C | 1 |
| ATL | Bifunctional autolysin | 1 |
| CATA | Catalase | 1 |
| DEOC2 | Deoxyribose-phosphate aldolase 2 | 1 |
| FTHS | Formate-tetrahydrofolate ligase | 3 |
| G3P1 | Glyceraldehyde-3-phosphate dehydrogenase 1 | 1 |
| GPMA | 2,3-bisphosphoglycerate-dependent phosphoglycerate mutase | 1 |
| LIP1 | Lipase 1 | 6 |
| OAT2 | Ornithine aminotransferase 2 | 1 |
| PCKA | Phosphoenolpyruvate carboxykinase [ATP] | 1 |
| PGK | Phosphoglycerate kinase | 1 |
| PLC | 1-phosphatidylinositol phosphodiesterase | 4 |
| PTAS | Phosphate acetyltransferase | 1 |
| RL14 | 50S ribosomal protein L14 | 1 |
| RS8 | 30S ribosomal protein S8 | 2 |
| SSPA | Glutamyl endopeptidase | 2 |
| SSPB | Staphopain B | 4 |
|  | | |
| **CH5/ph1** | | |
| CODY | GTP-sensing transcriptional pleiotropic repressor CodY 1 | 1 |
| ETXD | Enterotoxin type D | 2 |
| HLA | Alpha-hemolysin | 6 |
| LIP1 | Lipase 1 | 12 |
| LIP2 | Lipase 2 | 10 |
| MQO2 | Probable malate:quinone oxidoreductase 2 | 1 |
| PLC | 1-phosphatidylinositol phosphodiesterase | 3 |
| TKT | Transketolase | 1 |
| Y2979 | N-acetylmuramoyl-L-alanine amidase domain-containing protein SAOUHSC_02979 | 6 |
| **ph1/CH5** | | |
| ADH | Alcohol dehydrogenase | 1 |
| CATA | Catalase | 1 |
| ENO | Enolase | 3 |
| FTHS | Formate-tetrahydrofolate ligase | 2 |
| PCKA | Phosphoenolpyruvate carboxykinase [ATP] | 2 |
| PLC | 1-phosphatidylinositol phosphodiesterase | 1 |
| PTHP | Phosphocarrier protein HPr | 2 |
| SSPA | Glutamyl endopeptidase | 7 |
|  | | |
| **CH9/ch22** | | |
| FTHS | Formate-tetrahydrofolate ligase | 2 |
| HLA | Alpha-hemolysin | 1 |
| PCKA | Phosphoenolpyruvate carboxykinase [ATP] | 3 |
| PLS | Putative surface protein SAV2496/SAV2497 | 6 |
| SASG | Surface protein G | 6 |
| **ch22/CH9** | | |
| AHPC | Alkyl hydroperoxide reductase subunit C | 1 |
| LIP2 | Lipase 2 | 1 |
| LTAS | Lipoteichoic acid synthase | 1 |
| ODPA | Pyruvate dehydrogenase E1 component subunit alpha | 1 |
| SDRE | Serine-aspartate repeat-containing protein E | 2 |
|  | | |
| **CH9/ph2** | | |
| ATL | Bifunctional autolysin | 2 |
| CATA | Catalase | 1 |
| FTHS | Formate-tetrahydrofolate ligase | 2 |
| HLA | Alpha-hemolysin | 1 |
| NUC | Thermonuclease | 1 |
| PCKA | Phosphoenolpyruvate carboxykinase [ATP] | 2 |
| PLS | Putative surface protein SAV2496/SAV2497 | 3 |
| SASG | Surface protein G | 1 |
| **ph2/CH9** | | |
| AHPC | Alkyl hydroperoxide reductase subunit C | 1 |
| ATL | Bifunctional autolysin | 1 |
| CATA | Catalase | 1 |
| FTHS | Formate-tetrahydrofolate ligase | 1 |
| PGK | Phosphoglycerate kinase | 1 |
| SSPA | Glutamyl endopeptidase | 1 |
|  | | |
| **CH23/ch22** | | |
| ADH | Alcohol dehydrogenase | 1 |
| AHPC | Alkyl hydroperoxide reductase subunit C | 1 |
| CLFB | Clumping factor B | 1 |
| **ch22/CH23** | | |
| ODPA | Pyruvate dehydrogenase E1 component subunit alpha | 1 |
| SDRE | Serine-aspartate repeat-containing protein E | 3 |
|  | | |
| **CH23/ph2** | | |
| ATL | Bifunctional autolysin | 3 |
| CLFB | Clumping factor B | 1 |
| HLA | Alpha-hemolysin | 6 |
| **ph2/CH23** | | |
| ADH | Alcohol dehydrogenase | 1 |
| CATA | Catalase | 1 |
| CATB | Catalase-like protein | 1 |
| ENO | Enolase | 1 |
| G3P1 | Glyceraldehyde-3-phosphate dehydrogenase 1 | 2 |
| HCHA | Molecular chaperone Hsp31 and glyoxalase 3 1 | 1 |
| PGK | Phosphoglycerate kinase O | 1 |
| SSPA | Glutamyl endopeptidase | 5 |
| SSPB | Staphopain B | 3 |
|  | | |
| **PA2/ch22** | | |
| ADH | Alcohol dehydrogenase | 1 |
| FTHS | Formate-tetrahydrofolate ligase | 2 |
| GPMA | 2,3-bisphosphoglycerate-dependent phosphoglycerate mutase | 1 |
| PCKA | Phosphoenolpyruvate carboxykinase [ATP] | 2 |
| PLS | Putative surface protein SAV2496/SAV2497 | 11 |
| SASG | Surface protein G | 8 |
| **ch22/PA2** | | |
| G3P1 | Glyceraldehyde-3-phosphate dehydrogenase 1 | 1 |
| LIP2 | Lipase 2 | 3 |
| ODPB | Pyruvate dehydrogenase E1 component subunit beta | 2 |
| SDRE | Serine-aspartate repeat-containing protein E | 4 |
| SSPA | Glutamyl endopeptidase | 2 |
|  | | |
| **PA2/ph2** | | |
| ATL | Bifunctional autolysin | 1 |
| HLA | Alpha-hemolysin | 1 |
| PCKA | Phosphoenolpyruvate carboxykinase [ATP] | 1 |
| PLS | Putative surface protein SAV2496/SAV2497 | 3 |
| SASG | Surface protein G | 5 |
| **ph2/PA2** | | |
| AHPC | Alkyl hydroperoxide reductase subunit C | 2 |
| ENO | Enolase | 2 |
| FTHS | Formate-tetrahydrofolate ligase | 1 |
| PGK | Phosphoglycerate kinase | 1 |
| SDRE | Serine-aspartate repeat-containing protein E | 2 |
| SSPA | Glutamyl endopeptidase | 7 |
| SSPB | Staphopain B | 2 |

**S2 Table. A full list of proteins differentiating in pairs VIR/NVIR strain with MS data.**

Exel file: s4_table.xls

**S3 Table. A list of proteins identified as up-regulated in VIR strains.**

| Protein name | Acronym | Strain pairs where the protein was identified with the number of identifications in each pair |
| --- | --- | --- |
| **Bifunctional autolysin** | **ATL** | **CH3/pa3; 1**  **CH3/ch24; 1**  **CH5/ch24; 1**  **CH9/ph2; 2**  **CH23/ph2; 3**  **PA2/ph2; 1** |
| Alpha-hemolysin | HLA | CH3/ch24; 8  CH3/ pa3; 3  CH3/ ph1; 5  CH5/ch24; 5  CH5/ pa3; 7  CH5/ ph1; 6  CH9/ch22; 1  CH9/ph2; 1  CH23/ph2; 6  PA2/ph2;1 |
| **Formate-tetrahydrofolate ligase** | **FTHS** | **CH3/ph1; 1**  **CH5/pa3; 1**  **CH9/ch22; 2**  **CH9/ph2; 2**  **PA2/ch22;2** |
| **Phosphoenolpyruvate carboxykinase [ATP]** | **PCKA** | **CH3/ph1; 1**  **CH5/pa3; 1**  **CH9/ch22; 3**  **CH9/ph2; 2**  **PA2/ch22; 2**  **PA2/ph2; 1** |
| **Alcohol dehydrogenase** | **ADH** | **CH23/ch22; 1**  **PA2/ch22; 1** |
| **Catalase** | **CATA** | **CH5/pa3; 1**  **CH9/ph2; 1** |
| ATP-dependent Clp protease ATP-binding subunit ClpL | CLPL | CH3/ch24; 1  CH5/ch24; 2  CH5/pa3; 2 |
| Enterotoxin type D | ETXD | CH3/ch24; 4  CH3/ pa3; 5  CH3/ ph1; 4  CH5/ch24; 2  CH5/ pa3; 2  CH5/ ph1; 2 |
| **Lipase 1** | **LIP1** | **CH3/ch24; 7**  **CH3/ pa3; 3**  **CH3/ ph1; 6**  **CH5/ch24; 6**  **CH5/ pa3; 12**  **CH5/ ph1;** **12** |
| **Lipase 2** | **LIP2** | **CH3/ch24; 5**  **CH3/ pa3; 3**  **CH3/ ph1; 11**  **CH5/ch24; 4**  **CH5/ pa3; 14**  **CH5/ ph1; 10** |
| Probable malate:quinone oxidoreductase 2 | MQO2 | CH3/ph1; 1  CH5/ph1; 1 |
| **Thermonuclease** | **NUC** | **CH3/ch24; 1**  **CH9/ph2; 1** |
| **1-phosphatidylinositol phosphodiesterase** | **PLC** | **CH3/ch24; 3**  **CH3/ph1; 3**  **CH5/ch24; 2**  **CH5/ph1; 3** |
| **Putative surface protein SAV2496/SAV2497** | **PLS** | **CH9/ch22; 6**  **CH9/ph2; 3**  **PA2/ch22; 11**  **PA2/ph2; 3** |
| Surface protein G | SASG | CH9/ch22; 6  CH9/ph2; 1  PA2/ch22; 8  PA2/ph2;5 |
| **Serine-aspartate repeat-containing protein E** | **SDRE** | **CH3/ch24; 1**  **CH5/ch24; 1**  **CH5/pa3; 3** |
| Transketolase | TKT | CH3/ch24; 1  CH5/ph1; 1 |
| **Putative universal stress protein SA1532** | **Y1532** | **CH3/ph1; 1**  **CH5/pa3; 1** |
| N-acetylmuramoyl-L-alanine amidase domain-containing protein SAOUHSC_02979 | Y2979 | CH3/ch24; 2  CH3/ph1; 5  CH5/ ch24; 3  CH5/pa3; 4  CH5/ph1; 6 |
| **Alkyl hydroperoxide reductase subunit C** | **AHPC** | **CH23/ch22; 1** |
| **Fructose-bisphosphate aldolase** | **ALF2** | **CH5/pa3; 1** |
| Beta-lactamase | BLAC | CH5/ch24; 1 |
| Diacetyl reductase [(S)-acetoin forming] | BUTA | CH5/pa3; 1 |
| Clumping factor B | CLFB | CH23/ch22; 1  CH23/ph2; 1 |
| GTP-sensing transcriptional pleiotropic repressor CodY | CODY | CH5/ph1; 1 |
| Dihydrolipoyl dehydrogenase | DLDH | CH3/ph1; 1 |
| Chaperone protein DnaK | DNAK | CH5/pa3; 1 |
| Elongation factor G | EFG | CH5/pa3; 1 |
| **Elongation factor Tu** | **EFTU** | **CH5/ch24; 1** |
| **Glyceraldehyde-3-phosphate dehydrogenase 1** | **G3P1** | **CH3/ph1; 1** |
| **2,3-bisphosphoglycerate-dependent phosphoglycerate mutase** | **GPMA** | **PA2/ch22; 1** |
| Urocanate hydratase | HUTU | CH5/pa3; 1 |
| **Probable transglycosylase isaA** | **ISAA** | **CH3/ch24; 1** |
| Ribose-phosphate pyrophosphokinase | KPRS | CH5/ch24; 1  CH5/pa3; 1 |
| Pyruvate kinase | KPYK | CH5/pa3; 2 |
| Uncharacterized leukocidin-like protein 1 | LUKL1 | CH3/pa3; 2 |
| Formate acetyltransferase | PFLB | CH5/pa3; 2 |
| Polyribonucleotide nucleotidyltransferase | PNP | CH5/pa3; 1 |
| Immunoglobulin G-binding protein A | SPA | CH3/ch24; 1 |
| Serine protease splA | SPLA2 | CH3/ch24; 1 |
| Serine protease splB | SPLB | CH3/ch24; 1 |
| Glutamate--tRNA ligase | SYE | CH5/pa3; 1 |
| Threonine--tRNA ligase | SYT | CH5/pa3; 1 |
| Thioredoxin reductase | TRXB | CH5/pa3; 1 |
| UPF0355 protein MRSA252 | UP355 | CH3/ph1; 1 |
| UPF0447 protein SAR0593 | Y593 | CH5/pa3; 1 |

^a^ Proteins identified as differentiating in both VIR and NVIR strain proteomes are bolded.

**S4 Table. A list of proteins identified as up-regulated in NVIR strains.**

| Protein name | Acronym | Strain pairs where the protein was identified with the number of identifications in each pair |
| --- | --- | --- |
| Glutamyl endopeptidase | SSPA | ch22/PA2; 2  ch24/CH5; 3  pa3/CH5; 2  ph1/CH3; 2  ph1/CH5; 7  ph2/CH9; 1  ph2/CH23; 5  ph2/PA2; 7 |
| **Alcohol dehydrogenase** | **ADH** | **ch24/CH5; 1**  **pa3/CH5; 1**  **ph1/CH5; 1**  **ph2/CH23; 1** |
| **Alkyl hydroperoxide reductase subunit C** | **AHPC** | **ch22/CH9; 1**  **ch24/CH3; 2**  **ch24/CH5; 3**  **pa3/CH5; 1**  **ph2/CH9; 1**  **ph2/PA2; 2** |
| Enolase | ENO | ch24/CH5; 1  pa3/CH3; 1  ph1/CH3; 1  ph1/CH5; 3  ph2/CH23; 1  ph2/PA2; 2 |
| **Bifunctional autolysin** | **ATL** | **ch24/CH3; 1**  **ch24/CH5; 1**  **pa3/CH5; 1**  **ph2/CH9; 1** |
| **Catalase** | **CATA** | **pa3/CH5; 1**  **ph1/CH5; 1**  **ph2/CH9; 1**  **ph2/CH23; 1** |
| **Glyceraldehyde-3-phosphate dehydrogenase 1** | **G3P1** | **ch22/PA2; 1**  **pa3/CH5; 1**  **ph2/CH23; 2** |
| **Phosphoenolpyruvate carboxykinase [ATP]** | **PCKA** | **ch24/CH3; 2**  **ch24/CH5; 2**  **pa3/CH3; 2**  **pa3/CH5; 1**  **ph1/CH3; 2**  **ph1/CH5; 2** |
| **1-phosphatidylinositol phosphodiesterase** | **PLC** | **ch24/CH3; 1**  **ch24/CH5; 1**  **pa3/CH3; 3**  **pa3/CH5; 4**  **ph1/CH3; 1**  **ph1/CH5; 1** |
| Staphopain B | SSPB | ch24/CH3; 2  ch24/CH5; 3  pa3/CH5; 4  ph2/CH23; 3  ph2/PA2; 2 |
| **Lipase 1** | **LIP1** | **ch24/CH3; 2**  **ch24/CH5; 1**  **pa3/CH3; 2**  **pa3/CH5; 6** |
| Collagen adhesin | CNA | ch24/CH3; 3  ch24/CH5; 4  ph1/CH3; 1 |
| 3-hydroxyacyl-[acyl-carrier-protein] dehydratase FabZ | FABZ | ch24/CH5; 1  ph1/CH3; 1 |
| **Formate--tetrahydrofolate ligase** | **FTHS** | **pa3/CH3; 1**  **pa3/CH5; 3**  **ph1/CH5; 2**  **ph2/CH9;1**  **ph2/PA2;1** |
| Molecular chaperone Hsp31 and glyoxalase 3 | HCHA | ch24/CH5; 1  ph2/CH23; 1 |
| **Probable transglycosylase IsaA** | **ISAA** | **ch24/CH5; 1**  **pa3/CH3; 1** |
| Pyruvate dehydrogenase E1 component subunit beta | ODPB | ch22/PA2; 2  ch24/CH5; 2 |
| Phosphoglycerate kinase | PGK | pa3/CH5; 1  ph2/CH9; 1  ph2/CH23; 1  ph2/PA2; 1 |
| **Serine-aspartate repeat-containing protein E** | **SDRE** | **ch22/CH9; 2**  **ch22/CH23; 3**  **ch22/PA2;**  **ph2/PA2; 2** |
| Staphopain A | SSPP | pa3/CH3; 1  ph1/CH3; 1 |
| Acetate kinase | ACKA | ch24/CH5; 1 |
| Fructose-bisphosphate aldolase class 1 | ALF1 | ch24/CH5; 1 |
| **Fructose-bisphosphate aldolase** | **ALF2** | **ch24/CH5; 1** |
| Catalase-like protein | CATB | ph2/CH23; 1 |
| 10 kDa chaperonin | CH10 | ch24/CH3; 1 |
| Clumping factor A | CLFA | ph1/ CH3; 1 |
| Cysteine synthase | CYSK | ch24/CH3; 2  ch24/CH5; 2 |
| Deoxyribose-phosphate aldolase 2 | DEOC2 | pa3/CH5; 1 |
| Alanine dehydrogenase 2 | DHA2 | ch24/CH3; 1  ch24/CH5; 3 |
| Elongation factor Ts | EFTS | ch24/CH5; 1 |
| **Elongation factor Tu** | **EFTU** | **ch24/CH3; 1** |
| Enterotoxin type H | ETXH | ch24/CH3; 1  ch24/CH5; 11 |
| Glucose-6-phosphate isomerase | G6PI | ch24/CH5; 1 |
| Phosphoribosyl aminoimidazole carboxylase, catalytic subunit | gi|87162294 | ph1/CH3; 1 |
| **2,3-bisphosphoglycerate-dependent phosphoglycerate mutase** | **GPMA** | **pa3/CH5; 1** |
| Gamma-hemolysin component A | HLGA | ch24/CH3; 1 |
| 3-hexulose-6-phosphate synthase | HPS | ch24/CH5; 1 |
| Inosine-5'-monophosphate dehydrogenase | IMDH | ch24/CH3; 1  ch24/CH5; 1 |
| 6-phosphofructokinase | K6PF | ch24/CH5; 2 |
| L-lactate dehydrogenase 1 | LDH1 | ch24/CH5; 1 |
| **Lipase 2** | **LIP2** | **ch22/CH9; 1**  **ch22/PA2; 3** |
| Lipoteichoic acid synthase | LTAS | ch22/CH9; 1 |
| Molybdenum cofactor biosynthesis protein B | MOAB | ph1/ CH3; 1 |
| **Thermonuclease** | **NUC** | **pa3/CH3; 1** |
| Ornithine aminotransferase 2 | OAT2 | pa3/CH5; 1 |
| Pyruvate dehydrogenase E1 component subunit alpha | ODPA | ch22/CH9; 1  ch22/CH23; 1 |
| **Putative surface protein SAV2496/SAV2497** | **PLS** | **ch24/CH5; 1** |
| Putative peptidyl-prolyl cis-trans isomerase | PPI1 | pa3/CH3; 1 |
| Phosphate acetyltransferase | PTAS | pa3/CH5; 1 |
| Phosphocarrier protein HPr | PTHP | ph1/ CH5; 2 |
| 50S ribosomal protein L14 | RL14 | pa3/CH5; 1 |
| 50S ribosomal protein L5 | RL5 | pa3/CH3; 1 |
| 30S ribosomal protein S8 | RS8 | pa3/CH5; 2 |
| Superoxide dismutase [Mn/Fe] 1 | SODM1 | ch24/CH3; 1  ch24/CH5; 1 |
| Prophage-derived single-stranded DNA-binding protein | SSBP | ph1/CH3; 1 |
| Phenylalanine--tRNA ligase alpha subunit | SYFA | ch24/CH5; 2 |
| Signal transduction protein TRAP | TRAP | ph1/CH3; 1 |
| Uracil phosphoribosyltransferase | UPP | ch24/CH3; 1 |
| Uncharacterized N-acetyltransferase SA1019 | Y1019 | ch24/CH3; 1 |
| **Putative universal stress protein SA1532** | **Y1532** | **pa3/CH3; 2** |
| UPF0173 metal-dependent hydrolase SAB1566c | Y1566 | ch24/CH3; 1 |
| Uncharacterized protein SA1692 | Y1692 | ch24/CH5; 1 |
| Uncharacterized oxidoreductase SAS2370 | Y2370 | ch24/CH3; 1 |
| Uncharacterized protein SA0829 | Y829 | ch24/CH5; 1 |
| UPF0477 protein SA0873 | Y873 | ch24/CH5; 1 |
| Uncharacterized protein SAOUHSC_00997 | Y997 | ch24/CH3; 1  ch24/CH5; 2 |

^a^ Proteins identified as differentiating in both VIR and NVIR strain proteomes are bolded.

| **(name of the gel)** compared strains | | |
| --- | --- | --- |
| **(A)** pa3/CH5 | **(B)** ph2/CH23 | **(C)** ph2/PA2 |
| **name of the protein,** **form of the protein,** score; number of identified peptides; sequence coverage | | |
| **SSPA Z** 144.8; 3; 9.8 | **SSPA Z** 456.9; 10; 42.1 | **SSPA M** 820.8; 9; 45.9 |
|  |  |  |
| **SSPA Z** 855; 21; 62.8 | **SSPA M** 1614.6; 19; 55.6 | **SSPA M** 1003.5; 13; 56.6 |
|  |  |  |
| **SSPB Z** 1612.8; 22; 56 | **SSPA M** 1306.2; 13; 44 | **SSPA M** 372.2; 7; 35.4 |
|  |  |  |
| **SSPB Z** 2207.4; 26; 66.4 | **SSPA D**  545.3; 7; 28.1 | **SSPA D** 305.9; 5; 25.4 |
|  |  |  |
| **SSPB Z** 2178.9; 27; 59.5 | **SSPA D** 906.2; 9; 34.6 | **SSPA D** 530.9; 8; 27 |
|  |  |  |
|  | **SSPB M** 885.7; 10; 35.4 | **SSPA D** 364.7; 7; 31.8 |
|  |  |  |
|  | **SSPB M** 517.9; 8; 34.9 | **SSPA D** 304.2; 7; 31.8 |
|  |  |  |
|  | **SSPB D** 364.6; 6; 28.2 |  |
|  |  |  |

**S1 Figure.** **Sequences of glutamylendopeptidase (SSPA) and staphopain B (SSPB), described in Fig. 3 in the main manuscript.** Peptides identified during MS analysis are marked in red.

| **(name of the gel), name of the virulent strain**  spot number; score; number of identified peptides; sequence coverage | **name of the non-virulent strain** spot number; score*; number of identified peptides; sequence coverage | |
| --- | --- | --- |
| **LIP1 (lipase1)** | | |
| **(A) CH3** 554; 1599.2; 25; 44.4 | **ch24**  553; 592; 12; 19.6 | |
|  |  | |
| **(B) CH3** 48; 3862.9; 55; 75.1 | **pa3** 62; 2493; 39; 51.3 | |
|  |  | |
| **(E) CH5** 236 ; 2365.8; 36; 58.2 | **pa3** 239; 1405.9; 24; 40.7 | |
|  |  | |
| **PLC (1-phosphatidylinositol phosphodiesterase)** | | |
| **(A) CH3** 544; 478.5, 10, 31.1 | **ch24** 540; 443.7; 6; 26.5 | |
|  |  | |
| **(C) CH3** 308; 233.1; 5; 31.7 | **ph1** 298; 277; 7; 14.4 | |
|  |  | |
| **(D) CH5** 399; 638.4; 10; 32.7 | **ch24** 405 ; 695.5; 12; 37.2 | |
|  |  | |
| **(F) CH5** 346; 478.5; 10; 31.1 | **ph1** 350; 428.2; 12; 45.8 | |
|  |  | |
| **FTHS (Formate-tetrahydrofolate ligase)** | | |
| **(E) CH5** 283; 2504; 35; 68.3 | | **pa3** 281; 2178; 31; 60.7 |
|  | |  |
| **PCKA (Phosphoenolpyruvate carboxykinase [ATP])** | | |
| **(E) CH5** 283; 3146.8; 46;76.8 | **pa3** 285; 1367.6; 24; 55.3 | |
|  |  | |
| **CATA (Catalase)** | | |
| **(E) CH5** 278; 186.4; 5; 9.5 | **pa3** 280; 1530.5; 25; 61.2 | |
|  |  | |
| **ATL ( Bifunctional autolysin)** | | |
| **(A) CH3** 534; 284.1; 3.8 | **ch24** 563; 716;16;12.7 | |
|  |  | |
| **(D) CH5** 404; 216.3; 4; 5.0 | **ch24** 410; 999.8; 13.1 | |
|  |  | |

**S2 Figure. Examples of sequences of proteins identified as non-overlapping with identical identifications in VIR and NVIR, marked in Fig. 4 in the main manuscript.** Peptides identified during MS analysis are marked in red.


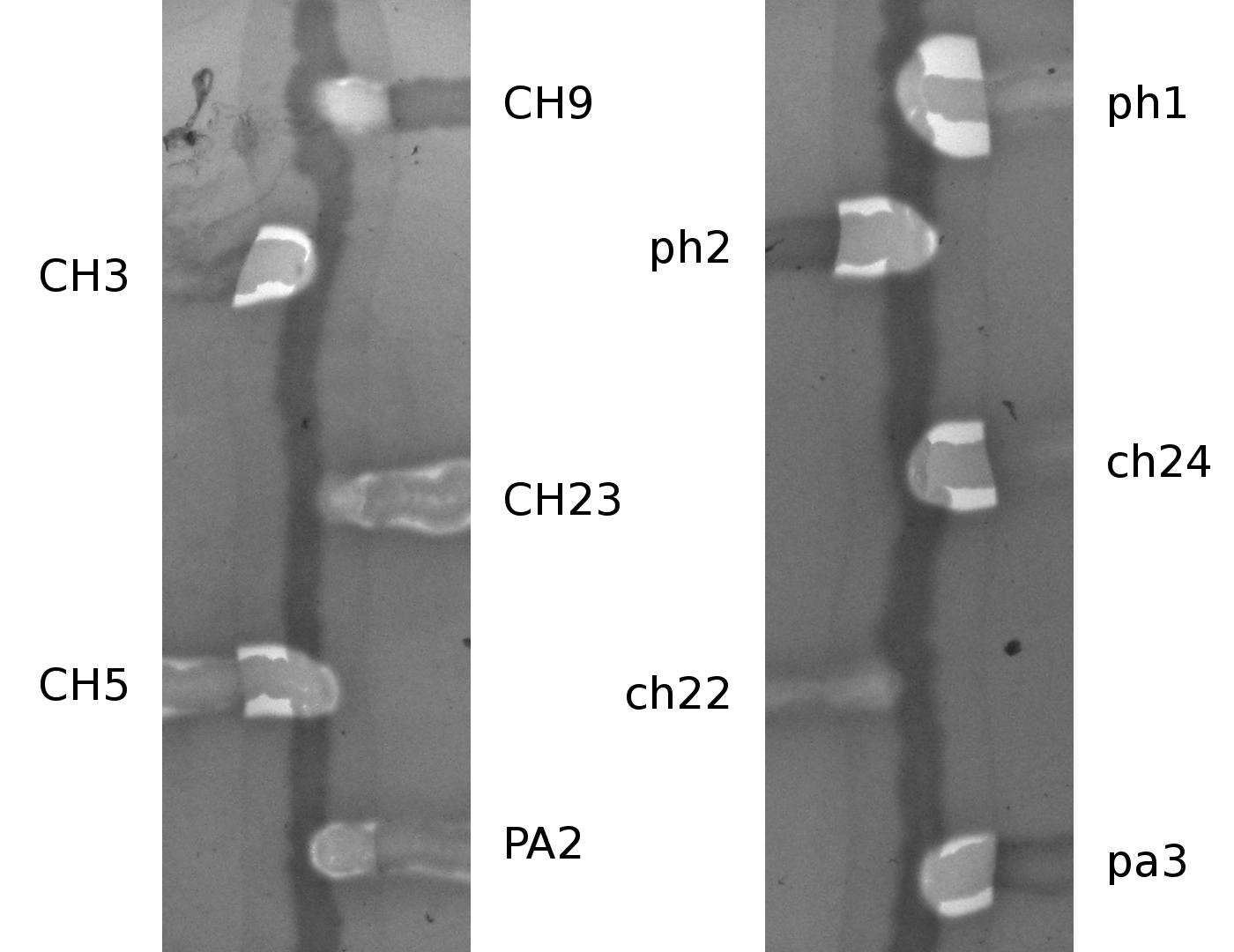


**S3 Figure. Delta-haemolysin assay.** The poultry-derived *S. aureus* strains were cross-streaked perpendicularly to *S. aureus* RN4220 (vertical streak), which produces only β-haemolysin, on a sheep blood agar plate. The enhanced zone of hemolysis is created by the interaction of the β-haemolysin of RN4420 and the delta-hemolysin of the test strain.
